# Single‐cell transcriptomics uncovers an instructive T‐cell receptor role in adult γδ T‐cell lineage commitment

**DOI:** 10.15252/embj.2021110023

**Published:** 2022-02-07

**Authors:** Sara Scaramuzzino, Delphine Potier, Robin Ordioni, Pierre Grenot, Dominique Payet‐Bornet, Hervé Luche, Bernard Malissen

**Affiliations:** ^1^ Centre d’Immunophénomique (CIPHE) Aix Marseille Université INSERM CNRS UMR Marseille France; ^2^ Centre d’Immunologie de Marseille‐Luminy (CIML) Aix Marseille Université INSERM CNRS Marseille France

**Keywords:** T cell, development, γδ T‐cell lineage specification, single‐cell transcriptomics, Immunology, Signal Transduction

## Abstract

After entering the adult thymus, bipotent T‐cell progenitors give rise to αβ or γδ T cells. To determine whether the γδ T‐cell receptor (TCR) has an instructive role in γδ T‐cell lineage commitment or only “confirms” a pre‐established γδ Τ‐cell lineage state, we exploited mice lacking expression of LAT, an adaptor required for γδ TCR signaling. Although these mice showed a T‐cell development block at the CD4^−^CD8^−^ double‐negative third (DN3) stage, 0.3% of their DN3 cells expressed intermediate levels of γδ TCR (further referred to as γδ^int^) at their surface. Single‐cell transcriptomics of LAT‐deficient DN3 γδ^int^ cells demonstrated no sign of commitment to the γδ T‐cell lineage, apart from γδ TCR expression. Although the lack of LAT is thought to tightly block DN3 cell development, we unexpectedly found that 25% of LAT‐deficient DN3 γδ^int^ cells were actively proliferating and progressed up to the DN4 stage. However, even those cells failed to turn on the transcriptional program associated with the γδ T‐cell lineage. Therefore, the γδ TCR‐LAT signaling axis builds upon a γδ T‐cell uncommitted lineage state to fully instruct adult γδ T‐cell lineage specification.

## Graphical abstract

TCR signaling via the LAT adaptor determines the developmental bifurcation of αβ and γδ T‐cell lineages in the adult mouse thymus.

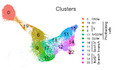

## Introduction

In mice, most T cells express a T‐cell antigen receptor (TCR) consisting of α and β chains, whereas a smaller population expresses an alternative form made of γ and δ chains. The TCR α, β, γ, and δ chains each contains a clonally variable (V) region that participates to antigen recognition. During intrathymic T‐cell development, the genes coding for TCR V regions assemble via site‐specific DNA recombinations that can result in productive, or in out‐of‐frame non‐productive rearrangements. Upon thymus colonization, T‐cell precursors develop into CD4^−^CD8^−^ double‐negative (DN) cells. Based on the expression of CD25 and CD44, DN cells can be organized according to the following developmental series: DN1 (CD44^+^CD25^−^) → DN2 (CD44^+^CD25^+^) → DN3 (CD44^−^CD25^+^) → DN4 (CD44^−^CD25^−^). DN3 cells simultaneously rearrange the *Trb, Trg,* and *Trd* genes that code for the TCR β, γ, and δ chains, respectively (Livak *et al*, 1999). Upon productive rearrangement of *Trg* and *Trd* genes, DN3 cells express a γδ TCR and in turn mature into CD4^−^CD8^−^ γδ T cells. Unlike conventional αβ T cells that acquire their effector function in the periphery, a large fraction of γδ T cells commit to an effector fate during intrathymic development. After migrating to the periphery and predominantly colonizing epithelial barriers, these “innate‐like” γδ T cells had the capacity to respond without delay to infection and tissue damage (Prinz *et al*, 2013; Hayday, 2019; Contreras & Wiest, 2020; Fiala *et al*, 2020; Parker & Ciofani, 2020; Hosokawa & Rothenberg, 2021). Upon productive *Trb* gene rearrangements, DN3 cells produce TCRβ chains that associate with invariant pre‐TCR alpha (pTα) chains to give rise to a pre‐TCR (Dutta *et al*, 2021). Pre‐TCR^+^ DN3 cells develop into CD4^+^CD8^+^ double‐positive (DP) cells that rearrange *Tra* genes that code for TCRα chains. On productive *Tra* gene rearrangements and replacement of pTα by TCRα, the resulting TCR αβ^+^ DP cells undergo positive and negative selection to generate CD4^+^ and CD8^+^ single‐positive (SP) cells that leave the thymus. γδ T‐cell development is thus punctuated by a single checkpoint termed γδ selection and operated by the γδ TCR, whereas αβ T‐cell development is subjected to two sequential checkpoints termed β and αβ selection and operated by the pre‐TCR and αβ TCR, respectively. Those checkpoints are intended to couple T‐cell development to the prior achievement of productive TCR gene rearrangements and the ensuing expression of αβ and γδ TCR. Developing T cells that fail crossing them are thus eliminated.

The pre‐TCR, αβ TCR, and γδ TCR associate with CD3 subunits that contain tyrosine‐based activation motifs. Upon phosphorylation by the LCK protein tyrosine kinase (PTK), these motifs recruit the PTK ZAP‐70 that in turn phosphorylates the adaptor protein LAT, resulting in the assembly of a LAT signalosome which mediates most of the TCR‐induced transcriptional responses (Mori *et al*, 2021). The requirement for pre‐TCR and γδ TCR signaling during early T‐cell development can be documented by the arrest of αβ and γδ T‐cell development at the DN3 stage in mice deficient in most CD3 subunits or LAT (Malissen *et al*, 1999; Munoz‐Ruiz *et al*, 2016). Two models have been proposed to explain how DN3 cells engage into the αβ or γδ T‐cell lineage (reviewed in Parker & Ciofani, 2020). The precommitment (or stochastic selective) model postulates that αβ versus γδ T‐cell lineage commitment is determined prior to and independently of the outcome of *Trb*, *Trg*, and *Trd* gene rearrangements. Accordingly, only those thymocytes whose predetermined lineage‐specific molecular “wiring” matches the distinct signals delivered by the stochastically expressed pre‐TCR or γδ TCR are able to further develop. In contrast, the instructive model of αβ versus γδ T‐cell lineage commitment posits that it is the distinct qualitative or quantitative characteristics of the pre‐TCR and γδ TCR signals received by DN3 cells that specify the αβ versus γδ lineage choice, respectively. The observation that a single transgenic γδ TCR was capable of triggering αβ lineage specification, upon attenuation of its signaling output (Haks *et al*, 2005; Hayes *et al*, 2005; Ciofani *et al*, 2006; Zhao *et al*, 2019), led to refine the instructive model into a signal strength model of αβ versus γδ T‐cell lineage commitment in which the pre‐TCR and γδ TCR were postulated to deliver weak and strong signals, respectively. In contrast, the fate of IL17‐producing (Tγδ17) γδ T cells that arise during fetal life appears determined prior to the onset of TCR gene rearrangement, suggesting that some γδ T‐cell subsets might develop according to a precommitment model (Melichar *et al*, 2007; Spidale *et al*, 2018).

Mouse T‐cell developmental stages are generally well distinguished by combinations of cell surface markers. However, individual T cells within a defined developmental stage might still display some heterogeneity reflecting different developmental potential, an issue that cannot be unveiled by “bulk” transcriptomic studies (Mingueneau *et al*, 2013; Roels *et al*, 2020). In contrast, single‐cell RNA sequencing (scRNAseq) permits to analyze genome‐wide RNA expression at single‐cell levels and to determine the degree of transcriptomics heterogeneity of a given T‐cell precursor population (Klein *et al*, 2019, Oh *et al*, 2021, Rothenberg, 2021, Sagar *et al*, 2020). Here, we used scRNAseq to analyze the developmental bifurcation leading to αβ and γδ T cells in the thymus of adult wild‐type (WT) and LAT‐deficient mice. It allowed us to assess whether γδ TCR signals build upon a “blank slate” to initiate γδ T–cell lineage specification or limit their role to “confirm” a γδ T–cell transcriptional choice made prior to *Trg* and *Trd* gene rearrangements. Moreover, it provided an unprecedented single‐cell resolution view of the commonalities and differences in the transcriptional signatures resulting from pre‐TCR, γδ TCR, and αβ TCR signaling at distinct stages of adult intrathymic development.

## Results

### scRNAseq analysis of the developmental bifurcation leading to αβ and γδ T cells in adult thymus

**Figure 1:**
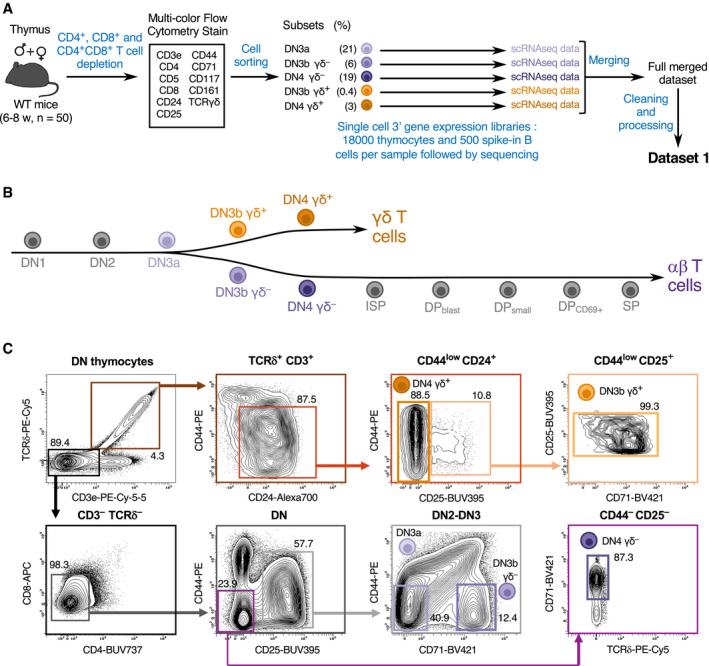
Schematic of scRNAseq experimental procedures Schematic representation of the workflow used for scRNAseq analysis of thymus and the generation of Dataset 1. Thymi were pooled from 6‐ to 8‐week‐old C57BL/6 female (*n* = 25) and male (*n* = 25) mice. Five single‐cell 3’ gene expression libraries containing 18,000 thymocytes and 500 spike‐in B cells were constructed and individually sequenced.Schematic representation of intrathymic T‐cell development stages. T‐cell subsets straddling the bifurcation leading to αβ and γδ T cells were sorted and are colored in purple or brown, respectively.Workflow used to sort the T‐cell subsets of interest. Thymocytes were first depleted from DP and SP T cells and then sorted by FACS. The sequential gating strategy used for sorting DN3a, DN3 γδ^+^, DN4 γδ^+^, DN3b γδ^−^, and DN4 γδ^−^ subsets is shown on the specified contour plots. CD71 expression was used to distinguish post‐β‐selected CD44^−^CD25^+^ DN3b γδ^−^ cells from unselected CD44^−^CD25^+^ DN3a cells. DN4 γδ^−^ cells constitute the progeny of DN3b γδ^−^ cells and were characterized by their CD71^+^CD44^+^CD25^−^ phenotype. The DN thymocytes gate corresponds to viable NK1.1^−^ cells. Among TCRδ^+^ CD3^+^ DN thymocytes, CD24^+^CD44^low^ cells correspond to DN3b γδ^+^ and DN4 γδ^+^ cells. DN3b γδ^+^ constitutes an intermediate stage bridging the DN3a and DN4 γδ^+^ stages, whereas DN4 γδ^+^ cells constituted the end product of the intrathymic γδ developmental pathway characterized here. TCRδ^+^ CD3^+^ DN thymocytes also comprise a minor subset of CD24^−^CD44^high^ cells (termed “cluster B”; (Prinz *et al*, 2006)) that contain NK‐like T cells. Numbers indicate percentages of cells found in each of the specified gates.

To investigate the developmental bifurcation leading to αβ and γδ T‐cell lineage specification at single‐cell resolution, DN cell subsets encompassing such bifurcation were isolated from 6‐ to 8‐week‐old thymus using established cell surface markers, and subjected to scRNAseq (Fig 1A). Considering that DN cells represent a quantitatively minor fraction of adult thymus, 50 WT thymi were harvested, pooled, and first depleted of DP and SP cells. Using multiparameter flow cytometry, the remaining DN cells were sorted into five subsets corresponding to DN3a cells, β‐selected αβ precursors (DN3b TCRγδ^−^ and DN4 TCRγδ^−^; in short DN3b γδ^−^ and DN4 γδ^−^), γδ‐selected γδ precursors (DN3b TCRγδ^+^; in short DN3b γδ^+^), and immature γδ T cells in the process of functional diversification (DN4 TCRγδ^+^; in short DN4 γδ^+^) (Fig 1B and C). To facilitate subsequent merging of the five individual scRNAseq datasets, 500 splenic B cells were added as an internal standard to each sorted thymocyte subset prior to subjecting them to scRNAseq (see Materials and Methods). After quality control of sequencing data, which included the removal of low‐quality cells expressing < 200 genes or expressing higher than 15% mitochondrial genes, the five scRNAseq datasets were merged into Dataset 1 (see Materials and Methods and Data Availability).

To visualize transcriptomic heterogeneity among Dataset 1 cells, we performed a non‐linear dimensionality reduction using Uniform Manifold Approximation and Projection (UMAP) method. The clusters corresponding to spike‐in B cells were identified in each sample based on *Cd74*, *Cd19*, and *Ms4a1* expression. Their positions fully overlapped, indicating no batch‐associated variability and precluding application of corrective measure to Dataset 1. After removing clusters corresponding to non‐T cells and to low‐quality cells showing a reduced expression of transcripts coding for the CD3δ and CD3ε subunits of the TCR‐CD3 complex, or a low percentage of ribosomal genes, the resulting Dataset 1 comprised of 57817 DN cells corresponding to 7520 DN3a, 11389 DN3b γδ^+^, 8010 DN4 γδ^+^, 18677 DN3b γδ^−^, and 12221 DN4 γδ^−^ cells.

### Single‐cell transcriptomics analysis of the earliest steps of γδ and αβ T‐cell commitment

**Figure 2:**
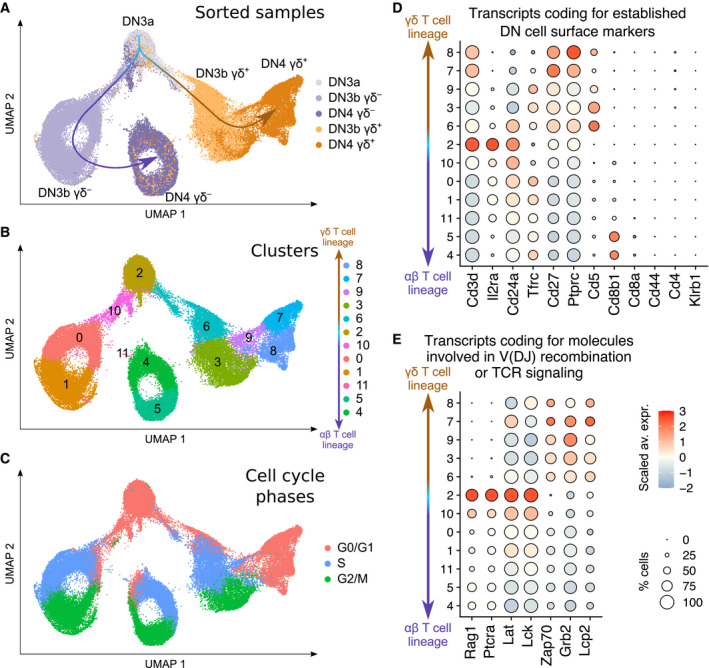
Single‐cell RNAseq profiling of WT DN3 and DN4 adult thymocytes A–CUMAP plots of Dataset 1 which contains five merged scRNAseq datasets corresponding to DN3b γδ^+^, DN4 γδ^+^, DN3a, DN3b γδ^−^, and DN4 γδ^−^ sorted cells and from which the small numbers of contaminating non‐T cells were excluded. Cell clustering was performed using a resolution of 0.4. The UMAP plots are colored according to the sorted cell samples (A), cell clusters (B), and cell cycle phases (C).D, EDot plots showing the expression level of transcripts coding for established markers of intrathymic T‐cell differentiation (D), and for molecules involved in V(D)J recombination and TCR signaling (E). In (D and E), dot color represents the scaled average expression of the specified gene across the various clusters, and dot size indicates the percentage of cells expressing the specified gene.

Congruent with current models of αβ and γδ T‐cell commitment (Parker & Ciofani, 2020), the UMAP plot corresponding to Dataset 1 showed two well‐separated branches emerging from DN3a cells (Fig 2A). One branch corresponded to the αβ T‐cell lineage and was made of DN3b γδ^−^ and DN4 γδ^−^ cells, whereas the other branch corresponded to the γδ T‐cell lineage and was made of DN3b γδ^+^ and DN4 γδ^+^ cells. Eleven cell clusters were identified by unsupervised analysis (Fig 2B; see Materials and Methods). DN3a cells mostly corresponded to a single cluster (cluster 2), whereas DN3b γδ^+^ and DN4 γδ^+^ cells comprised of two (3 and 6) and three (7, 8, and 9) clusters, respectively. DN3b γδ^−^ and DN4 γδ^−^ cells comprised of clusters 10, 0, and 1, and clusters 4, 5, and 11, respectively.

The γδ TCR‐induced DN3a → DN3b γδ^+^ transition is associated with a wave of cell proliferation (Prinz *et al*, 2006; Taghon *et al*, 2006), and most DN3b γδ^+^ cells were thus in the S (cluster 6) and G2/M (cluster 3) phases of the cell cycle and corresponded to transit‐amplifying cells (Fig 2C). The functional diversification that occurred in DN4 γδ^+^ cells (see below) was associated with a return to a non‐proliferative state (clusters 7, 8, and 9). As expected on the basis of former studies (Mingueneau *et al*, 2013), both DN3b γδ^−^ and DN4 γδ^−^ cells constituted transit‐amplifying cells. For instance, although a small fraction of DN3b γδ^−^ cells was in G0/G1 (cluster 10), their majority was in S (cluster 0) and G2/M (cluster 1) phases. Likewise, most DN4 γδ^−^ were in S (cluster 4) and G2/M (cluster 5) phases.

The analysis of well‐characterized cell surface markers of DN cell subsets validated our sorting strategy (Fig 2D). For instance, DN3a cells were the sole to express high levels of *Il2ra* which codes for CD25 (the interleukin 2 receptor α chain), and all the analyzed DN3‐DN4 subsets were negative for *Cd44* (a DN1‐DN2 cell marker) and *Klrb1* (a natural killer (NK) cell marker). *Cd8b1* that codes for the β chain of CD8αβ heterodimers started to be expressed in DN4 γδ^−^ that are the direct precursors of immature single‐positive (ISP) cells, an intermediate CD8^+^CD4^−^ stage bridging the DN4 and CD8^+^CD4^+^ DP stages. Consistent with their robust proliferation, DN3b γδ^+^, DN3b γδ^−^, and DN4 γδ^−^ cells expressed *Tfrc* transcripts that code for the transferrin receptor (also known as CD71; Kelly *et al*, 2007). As expected, expression of *Cd24* persisted in DN3b γδ^−^ and DN4 γδ^−^ cells and started decreasing in immature DN4 γδ^+^ prior to their differentiation into peripheral mature CD24^−^ TCRγδ^+^ cells (Parker & Ciofani, 2020). Finally, γδ selection triggered a stronger increase in the expression of *Cd5* and *Cd27* (that codes for TNFRSF7, a member of the tumor necrosis factor receptor superfamily) as compared to β selection.

Among the analyzed DN cell subsets, DN3a cells rearrange the *Trg*, *Trd*, and *Trb* genes and decode the first pre‐TCR or γδ TCR signals (Klein *et al*, 2019). They expressed the highest levels of transcripts coding for the RAG1 V(D)J recombinase subunit, the pTα chain (coded by *Ptcra*), the Src family protein tyrosine kinase (PTK) LCK, and the LAT transmembrane adaptor (Fig 2E). In contrast, when compared to γδ‐ and β‐selected cells, DN3a cells expressed the lowest levels of transcripts coding for ZAP‐70, which belongs to the Syk PTK family. This finding is consistent with the expression in DN3a cells of high levels of transcripts coding for the SYK PTK, which is the eponymous member of the Syk family and can substitute for ZAP‐70 (Muro *et al*, 2018). Moreover, as suggested by a former study (Palacios & Weiss, 2007), the increase in *Zap70* transcripts observed in DN3b γδ^−^ and DN4 γδ^−^ cells likely boosts pre‐TCR signals until the ISP stage is reached. Post‐selected DN3b γδ^+^ and DN4 γδ^+^ cells expressed the highest levels of transcripts coding for ZAP‐70 and for the intracytoplasmic adaptors SLP‐76 (encoded by *Lcp2*) and GRB2 among Dataset 1 cell clusters, likely contributing to reinforce the signaling strength of the γδ TCR as compared to that of the pre‐TCR.

### Functional diversification of adult DN4 γδ^+^ cells

**Figure 3:**
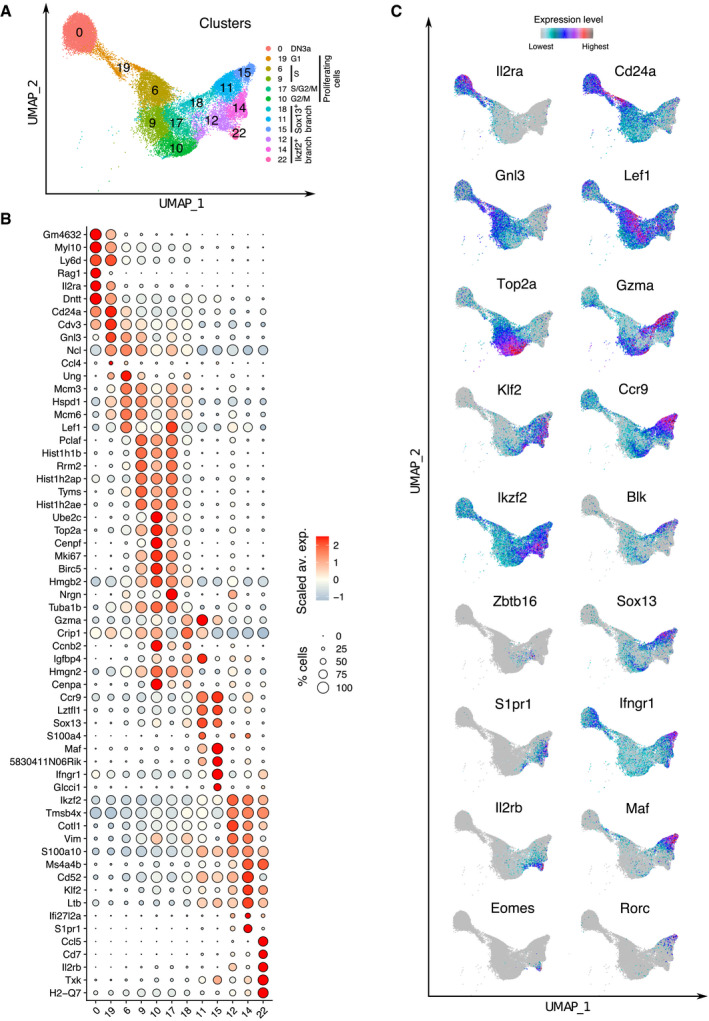
Intrathymic diversification of adult γδ T cells UMAP plots focusing on DN3a, DN3b γδ^+^, and DN4 γδ^+^ cells from Dataset 1. Clustering was performed using a 1.5 resolution. The UMAP plot is colored according to clusters and the main characteristics of the defined clusters specified in the right margin.Dot plot showing the expression level of the top 6 DEG markers characterizing each of the 12 clusters. Dot color represents the scaled average expression of the specified gene across the 12 clusters, and dot size indicates the percentage of cells expressing the specified gene.UMAP plots colored according to the expression level of the specified genes.

A recent scRNAseq‐based study compared γδ T‐cell functional diversification in fetal and adult thymus (Sagar *et al*, 2020). By zooming on the scRNAseq datasets corresponding to DN3a, DN3b γδ^+^, and DN4 γδ^+^ cells, we were able to explore further γδ T‐cell functional diversification in adult thymus (Fig 3A). Twelve cell clusters were identified among them using unsupervised hierarchical clustering with a fine‐grained resolution. DN3b γδ^+^ cells comprised of clusters 6, 9, 10, and 17, whereas DN4 γδ^+^ cells comprised of clusters 11, 12, 14, 15, 18, and 22. Using differential gene expression analysis, we identified the top 6 differentially expressed genes (DEG) markers of each cluster based on the best positive log fold change, and we organized them into a dot plot (Fig 3B).

Cluster 19 was contiguous to cluster 0 (DN3a cells), and the cells it contained (a mix of DN3a and DN3b γδ^+^ cells) started expressing *Gnl3* that codes for guanine nucleotide‐binding protein‐like 3 (GLN3 or nucleostemin; Fig 3B and C), suggesting their entry in the G1 phase of the cell cycle (Qu & Bishop, 2012). Consistent with our global analysis of Dataset 1 (Fig 2C), most of the remaining DN3b γδ^+^ clusters were in S or G2/M phase (Appendix Fig S1). Following such proliferative burst, the resulting DN4 γδ^+^ cells were predominantly resting and differentiated into IFN‐γ‐producing (Tγδ1) and Tγδ17 γδ T‐cell subsets. For instance, the expression of *Sox13,* a key transcription factor for Tγδ17 programming (Melichar *et al*, 2007), was first induced in cluster 18 which bridged cluster 19 of DN3b γδ^+^ cells to clusters 11 and 15 of DN4 γδ^+^ cells (Fig 3A). Both clusters 11 and 15 expressed *Blk* and *Maf*, two Tγδ17‐cell hallmarks (Zuberbuehler *et al*, 2019) in addition to *Sox13* (Fig 3B and C). Cluster 15 also expressed *Rorc* which codes for the nuclear receptor ROR gamma transcription factor that governs IL‐17 expression (Ivanov *et al*, 2006), whereas cluster 11 expressed *Gzma* and might correspond to a novel subset of granzyme A^+^ γδ T cells endowed with cytotoxic activity (Sagar *et al*, 2020).

The Tγδ1 functional branch was best characterized by the expression of high levels of *Ikzf2*, which codes for the zinc finger protein Helios, and it comprised three clusters (12, 14, and 22) (Fig 3A–C). Cluster 22 expressed *Il2rb* and *Eomes*, two hallmarks of the Tγδ1 cell subset. Conversely, *Il2rb* was not expressed in cluster 14 which instead expressed the sphingosine‐1‐phosphate receptor (*S1pr1*) that is critical for thymocyte egress (Carlson *et al*, 2006). Cluster 14 included a subset expressing *Ccr9*, a chemokine receptor involved in migration of γδ T cells to peripheral sites (Uehara *et al*, 2002). As recently suggested (Sagar *et al*, 2020), these *S1pr1* ^+^ *Ccr9* ^+^ cells likely correspond to a population of γδ T cells that leaves the thymus in a naive state and undergoes functional polarization in the periphery. Most cells of cluster 12 corresponded to a transitional cluster between DN3b γδ^+^ cells and the Tγδ1 clusters. The remaining cells found in cluster 12 expressed *Zbtb16* (that encodes PLZF, a lineage marker of NKT cells), suggesting that they are primed toward an IFN‐γ/IL4‐producing fate (Kreslavsky *et al*, 2009). Therefore, our fine‐grained scRNAseq analysis of DN4 γδ^+^ cells permitted to refine a recent model of adult intrathymic γδ^+^ T‐cell functional diversification (Sagar *et al*, 2020).

### Identifying the transcriptional signatures induced by the pre‐TCR and γδ TCR in adult thymus

**Figure 4:**
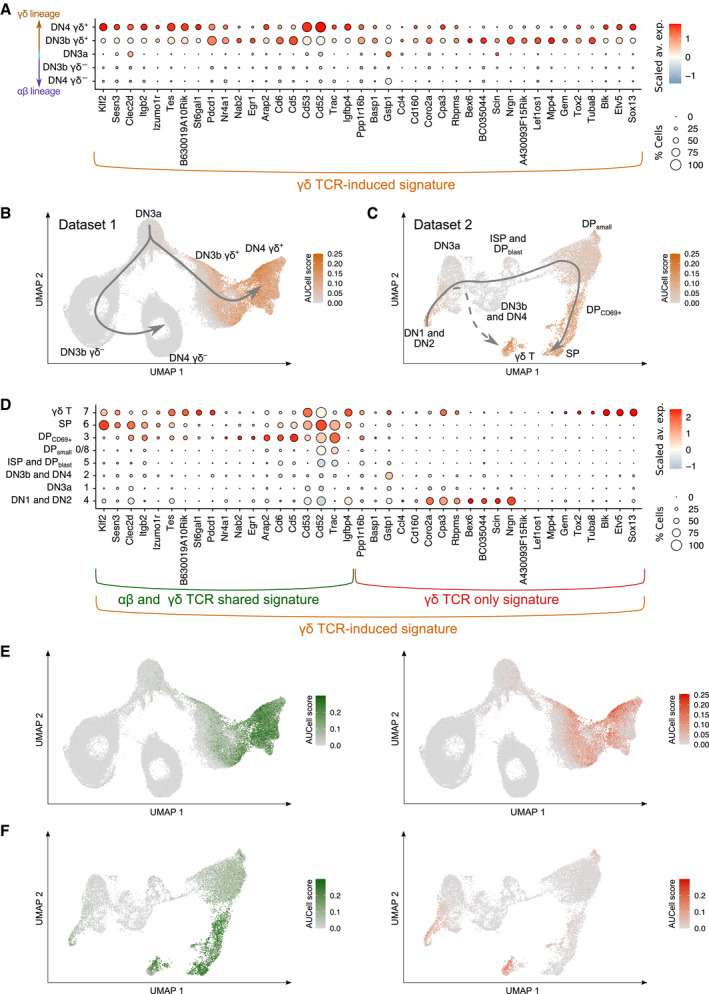
Transcriptional footprint of γδ T‐cell lineage commitment ADot plot showing the expression level of genes belonging to the γδ TCR‐induced signature across DN3a, DN3b γδ^+^, DN4 γδ^+^, DN3b γδ^−^, and DN4 γδ^−^ subsets of Dataset 1. The γδ TCR‐induced signature was defined by comparing the transcriptome of DN3b γδ^−^ and DN3b γδ^+^ cells (see Methods).B, CAUCell scores for the γδ TCR‐induced signature among the UMAP plot of cells corresponding to Dataset 1 (B) and Dataset 2 (C). As shown in Fig EV2, Dataset 2 corresponds to DN‐enriched scRNAseq data merged with whole thymus scRNAseq data.DDot plot showing the expression level of genes belonging to the γδ TCR‐induced signature across the cell subsets corresponding to Dataset 2. Based on the gene set that was specifically upregulated during the DP_small_ → DP_CD69_^+^/SP transition (see Fig EV3B), it allowed to subdivide the γδ TCR‐induced signature into a “γδ TCR only” signature and a “αβ and γδ TCR shared” signature.E, FAUCell scores for the “αβ and γδ TCR shared” signature (left panel) and for the “γδ TCR only” signature (right panel) among the UMAP plot of cells corresponding to Datasets 1 (E) and 2 (F).

**Figure EV1:**
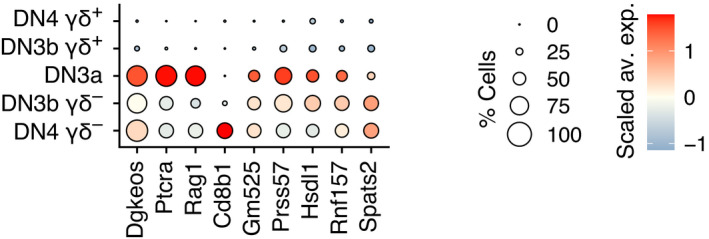
Transcriptional footprint of pre‐TCR selection Dot plot showing the expression level of genes characterizing the “pre‐TCR‐induced” signature among the DN3a, DN3b γδ^+^, DN4 γδ^+^, DN3b γδ^−^, and DN4 γδ^−^ sorted subsets of Dataset 1. The “pre‐TCR‐induced” signature was obtained by analyzing the genes differentially expressed between the DN3b γδ^−^/DN4 γδ^−^ and DN3b γδ^+^ sorted samples and by selecting the genes upregulated in DN3b γδ^−^/DN4 γδ^−^ subset (adjusted *P*‐value = 0 and expressed in < 25% of the DN3b γδ^+^ cells). A fully identical gene list was generated when a *P*‐value < 0.01 was used.

To characterize early γδ T‐cell specification, we identified those transcripts that were differentially expressed between the DN3b γδ^+^ and DN3b γδ^−^ cells. Transcripts linked to cell cycling and metabolic activity were excluded from such “γδ TCR‐induced signature” since most of them were induced in both the DN3b γδ^−^ and DN3b γδ^+^ transit‐amplifying cells. Likewise, most of the transcripts typifying DN3a cells were repressed at both the DN3a → DN3b γδ^+^ and DN3a → DN3b γδ^−^ transitions (Mingueneau *et al*, 2013; Rothenberg, 2019) and thus excluded from the γδ TCR‐induced signature, allowing to specifically focus on transcripts associated with early γδ T–cell specification. DEG with an adjusted *P*‐value of 0 and expressed in < 25% of the DN3b γδ^−^ cells were selected to generate the γδ TCR‐induced signature (Fig 4A). Using the AUCell tool (see Materials and Methods; Aibar *et al*, 2017), we identified which cells expressed the γδ TCR‐induced signature among the sorted cell subsets present in Dataset 1 (Fig 4B). As expected for a γδ TCR‐induced signature, DN3a cells showed the lowest AUCell scores, whereas high AUCell scores were only observed for DN3b γδ^+^ and DN4 γδ^+^ cells. To define a “pre‐TCR‐induced” signature, we took in consideration that the transit‐amplifying cells of the αβ T‐cell lineage branch comprised both DN3b γδ^−^ and DN4 γδ^−^ cells (Fig 2). Therefore, for the sake of symmetry, the genes that were differentially expressed between the DN3b γδ^−^/DN4 γδ^−^ cells and DN3b γδ^+^ cells were used to define the pre‐TCR‐induced signature and identify genes linked to early αβ T–cell specification (Fig EV1).

### Commonalities and differences in the transcriptional signatures induced by the pre‐TCR and γδ TCR in adult thymus

The pre‐TCR‐induced signature was narrower than the γδ TCR‐induced signature and limited to 10 genes (Fig EV1). Among them, *Cd8b1* transcripts that code for CD8β were the sole to be strongly induced during the DN3a → DN3b γδ^−^/DN4 γδ^−^ transition (Fig EV1). *Spats2* transcripts, which code for a protein involved in cell proliferation and called spermatogenesis‐associated serine‐rich protein (Dong *et al*, 2020), were also induced during this transition, although to a smaller extent than *Cd8b1* transcripts. The remaining eight genes of the pre‐TCR‐induced signature corresponded to genes that are expressed in DN3a cells and poorly repressed during the DN3a → DN3b γδ^−^/DN4 γδ^−^ transition as compared to the strong repression they are subject to during the DN3a → DN3b γδ^+^ transition. Some of them were fully repressed at the subsequent ISP stage (*Ptcra*), or reused in DP_small_ cells for *Tcra* gene rearrangements (*Rag1*) or signaling (*Dgkeos* transcripts coding for diacylglycerol kinase DGKε) (Mingueneau *et al*, 2013). Therefore, much of the transcriptional changes induced by both the pre‐TCR and γδ TCR corresponded to the shutdown of the DN3a program or to the upregulation of genes involved in the cell cycle or metabolic activity. Aside from this shared component, the remaining part of pre‐TCR‐ and γδ TCR‐induced transcriptional signatures markedly differed (Figs 4A and EV1). The former was primarily limited to the *Cd8b1* transcript, whereas the later consisted of a multitude of transcripts coding for proteins necessary for the functional maturation and the ensuing intrathymic differentiation of γδ T cells (*Klf2*, *Pdcd1, Cd5*, *Cd6*, *Cd52*, *Cd53*, *Cpa3*, *Blk, Etv5,* and *Sox13*).

### Identifying the transcriptional signatures induced by the αβ TCR in adult thymus

**Figure EV2:**
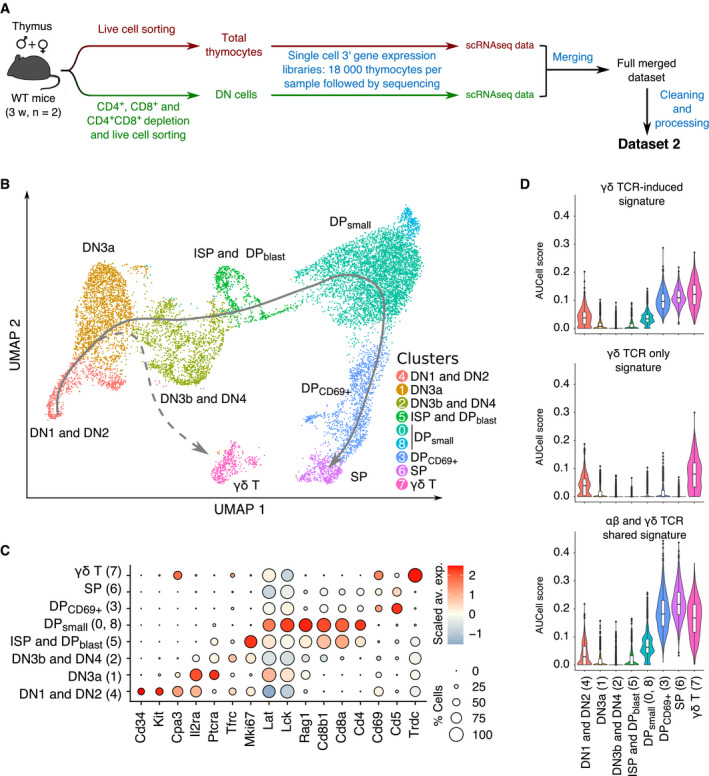
Expression of the γδ TCR‐induced signature across the whole sequence of intrathymic T‐cell development Schematic representation of the workflow used for scRNAseq analysis of total thymocytes and DN‐enriched thymocytes and the generation of Dataset 2.UMAP plots of the cells corresponding to Dataset 2 and colored according to the defined clusters.Dot plot showing the expression level of selected genes among the eight cell clusters identified in Dataset 2. Dot color represents the scaled average expression of the gene of interest across the various clusters, whereas dot size indicates the percentage of cells expressing the specified gene.Violin plots showing AUCell scores of the γδ TCR‐induced (top), γδ TCR‐only signature (middle), and the “αβ and γδ TCR shared” (bottom) signatures among the specified subsets.

**Figure EV3:**
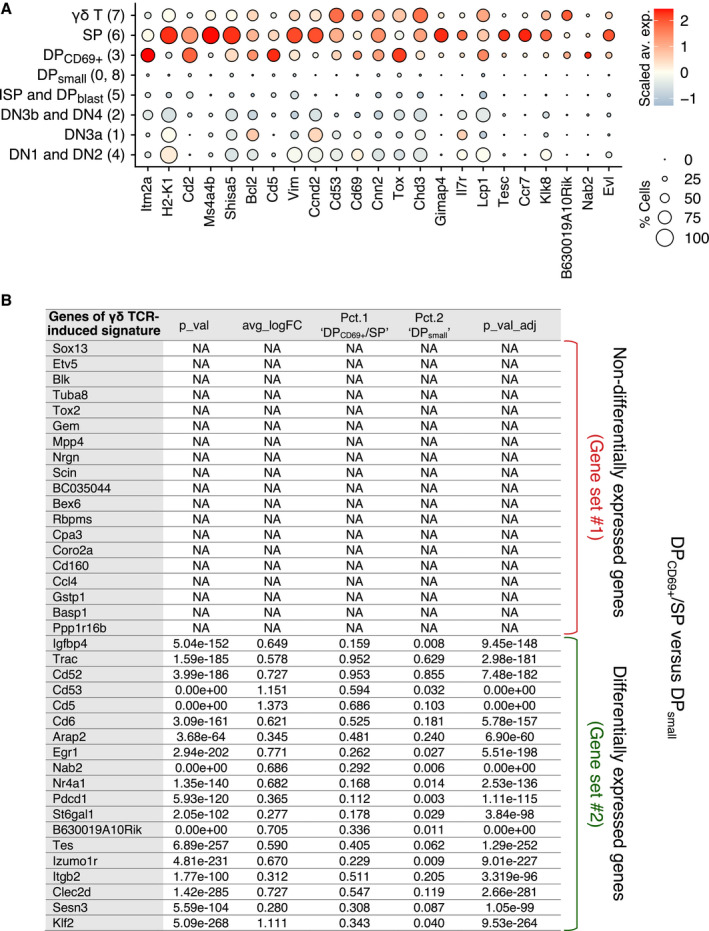
Analysis of the genes that are differentially expressed during the DP_small_ → DP_CD69+_/SP transition Dot plot showing the expression level of the top genes which are upregulated during the DP_small_ → DP_CD69+_/SP transition among the eight cell clusters identified in Dataset 2 (genes shown are expressed in < 25% of DP_small_ cell, have a *P*‐value = 0 and a log fold change > 0). Dot color represents the scaled average expression of the specified genes across the eight cell clusters, whereas dot size indicates the percentage of cells expressing the specified gene.The table summarized the results of differential gene expression analysis of DP_small_ → DP_CD69+_/SP transition using a Wilcoxon rank sum test (Seurat FindMarkers function used with default parameters). Only the genes corresponding to the γδ TCR‐induced signature are shown. The average log fold change (FC) corresponds to the log fold change of the average expression between the two groups. Positive values indicate that the gene is more highly expressed in the DP_CD69+_/SP group. The pct.1 DP_CD69+_/SP and pct.2 DP_small_ values correspond to the percentage of DP_CD69+_/SP and DP_small_ cells where the gene is detected, respectively. Adjusted *P*‐value (p_val_adj) is based on Bonferroni correction using all genes in the dataset. The genes not differentially expressed are denoted as “NA” (not available). Accordingly, the γδ TCR‐induced signature can be divided into two gene sets: gene set #1 consists of genes that are not upregulated in DP_CD69+_/SP cells and gene set #2 consists of genes upregulated in DP_CD69+_/SP cells. *P*‐values are indicated for both gene sets. Therefore, gene set #1 was defined as the “γδ TCR only” signature and gene set #2 as the “αβ and γδ TCR shared” signature.

To compare the γδ TCR‐induced transcriptional signature to that induced by the fully assembled αβ TCR at the DP_small_ → DP_CD69+_/SP transition, we generated two additional scRNAseq datasets. They corresponded to adult DN and total thymocytes that were each sorted for live cells, subjected to droplet‐based scRNA sequencing, and finally merged into Dataset 2 (Fig EV2A and see Materials and Methods). After controlling the quality of data merging, our clustering analysis identified nine clusters that were assigned to DN1/DN2, DN3a, DN3b/DN4, ISP/DP_blast_, DP_small_, DP_69+_, SP, and γδ T cells on the basis of markers commonly used to delineate adult thymic cell subsets (Fig EV2B and C). We used Dataset 2 to define DEG between the DP_CD69+_/SP cells and DP_small_ cells. Such “αβ TCR‐induced signature” comprised of transcripts coding for transmembrane receptors important for αβ T‐cell function (*Cd2*, *Cd5*, *Cd69*, and *Cd53*), and for differentiation (*Tox*) and survival (*Bcl2*) (Fig EV3A), consistent with a former bulk transcriptomics study (Mingueneau *et al*, 2013).

### Commonalities and differences in the transcriptional signatures induced by the γδ TCR and αβ TCR in adult thymus

When the γδ TCR‐induced signature was applied to Dataset 2 (Fig 4C), high AUCell scores were observed for both γδ T cells and DP_CD69+_/SP cells, suggesting that a part of the γδ TCR‐induced signature is also induced by the αβ TCR during the DP_small_ → DP_CD69+_/SP transition. The DP_small_ → DP_CD69+_/SP transition is not associated with proliferation (Mingueneau *et al*, 2013), facilitating the comparison of the αβ TCR‐induced signature with our γδ TCR‐induced signature which is pruned of genes involved in the cell cycle (Fig EV3B). It permitted to deconvolute the γδ TCR‐induced signature into a component expressed in the sole DN3b γδ^+^ cells (denoted as the “γδ TCR only signature”) and a component expressed in both DN3b γδ^+^ and DP_CD69+_/SP cells (denoted as the “γδ and αβ TCR shared signature”; Figs 4D–F and EV2D). The γδ TCR‐only signature comprised the *Etv5* and *Sox13* transcripts that code for transcription factors specifying the effector function of Tγδ17 cells, *Blk*, a Src family PTK implicated in Tγδ17‐cell development (Laird *et al*, 2010), and *Cpa3* which codes for carboxypeptidase A3 (Turchinovich & Hayday, 2011). The γδ and αβ TCR shared signature comprised of *Klf2*; *Sesn3,* coding for a member of the sestrin family of stress‐induced proteins; *Clec2d*, coding for a member of the natural killer cell receptor C‐type lectin family; *Itgb2*, coding for an integrin β chain also known as LFA‐1; *Tes*, coding for a scaffold protein playing a role in the actin cytoskeleton; *Cd53*, coding for a member of the transmembrane 4 superfamily; and *Cd52,* coding for CAMPATH‐1, as well as transcripts coding for the CD5 and CD6 transmembrane receptors which both play an important role in regulating TCR signals (Mori *et al* (2021); Fig EV3B). Therefore, apart from the genes involved in the cell cycle and in metabolic activity, the transcriptional signature induced by the γδ TCR at the DN3a → DN3b γδ^+^ transition was more similar to that induced by the αβ TCR at the DP_small_ → DP_CD69+_/SP transition than to that induced by the pre‐TCR.

### scRNAseq analysis of adult DN3 cells expressing signaling‐defective γδ TCR

**Figure EV4:**
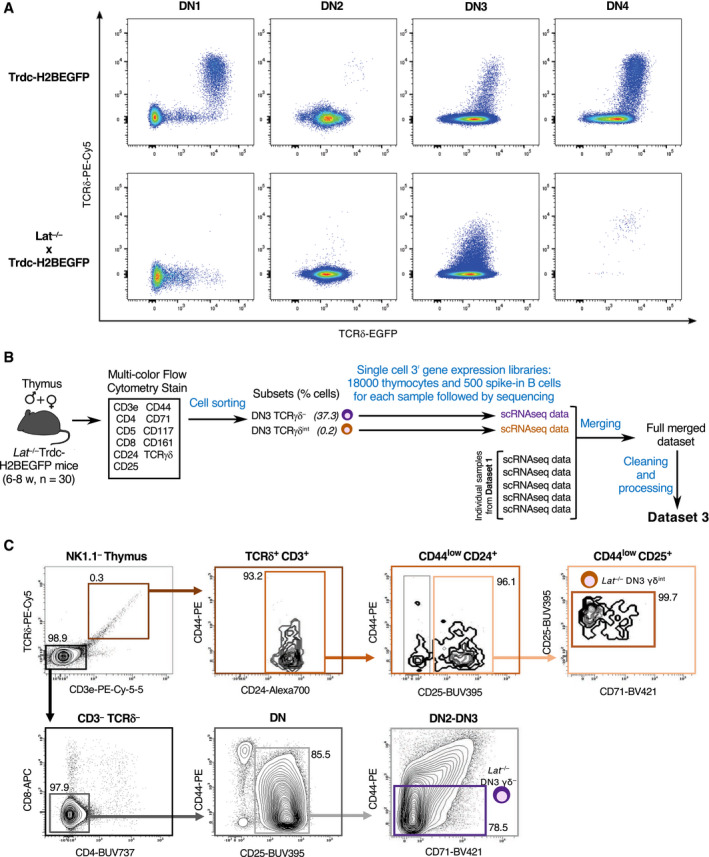
Workflow used for scRNAseq analysis of DN3 γδ^−^ and γδ^int^ cells from *Lat* ^−/−^ thymus Flow cytometry plots showing EGFP and surface TCR γδ expression in DN1, DN2, DN3, and DN4 subsets from *Trdc*‐*H2BEGFP* (top panels) and *Lat* ^−/−^ × *Trdc*‐*H2BEGFP* (bottom panels) mice. Note that the TCR γδ^+^ cells corresponding to the DN1 stage correspond to mature γδ T cells that present a CD127^hi^ phenotype and they were thus excluded from the analysis.Schematic representation of the workflow used for scRNAseq analysis of DN3 γδ^−^ and DN3 γδ^int^ cells from thymus (*n* = 30) of *Lat* ^−/−^ × *Trdc*‐*H2BEGFP* mice and the generation of Dataset 3. Two single‐cell 3’ gene expression libraries containing 18,000 thymoctes and 500 spike‐in B cells were constructed and individually sequenced.FACS plots showing the gating strategy used to sort DN3 γδ^−^ and DN3 γδ^int^ cells from *Lat* ^−/−^ × *Trdc*‐*H2BEGFP* mice. Numbers indicate percentages of cells found in each of the specified gates.

To support on the basis of genetic evidence the role of γδ TCR‐triggered signals in γδ T–cell lineage commitment and specification, we exploited *Trdc*‐*H2BEGFP* mice (Prinz *et al*, 2006). Those mice expressed an eGFP reporter gene under the control of the gene coding for the TCRδ chain, facilitating tracking of the earliest steps of γδ T‐cell lineage commitment. T‐cell development is blocked at the DN3 stage in *Trdc*‐*H2BEGFP* mice deficient for the LAT adaptor protein (*Lat* ^−/−^ x *Trdc*‐*H2BEGFP* mice) (Nunez‐Cruz *et al*, 2003), suggesting that LAT is required for both pre‐TCR and γδ TCR signaling. Approximately 0.3% of the DN3 cells found in *Lat* ^−/−^ x *Trdc*‐*H2BEGFP* thymus expressed intermediate level of γδ TCR at their surface (denoted in short as DN3 γδ^int^ cells; Fig EV4A), demonstrating that progression beyond such DN3 γδ^int^ stage is controlled by γδ TCR signals requiring the LAT adaptor (Prinz *et al*, 2006). Moreover, the lack of CD5 expression and of CD127 downregulation on those LAT‐deficient DN3 γδ^int^ cells suggested that at least part of the γδ T‐cell specification program was not induced in the absence of instructive γδ TCR signals (Prinz *et al*, 2006). Consistent with that view, infection of LAT‐deficient DN3 γδ^int^ cells with LAT‐expressing retrovirus induced the surface expression of CD5 and of high levels of γδ TCR as observed on a fraction of WT DN3b γδ^+^ cells (Muro *et al*, 2019).

**Figure 5:**
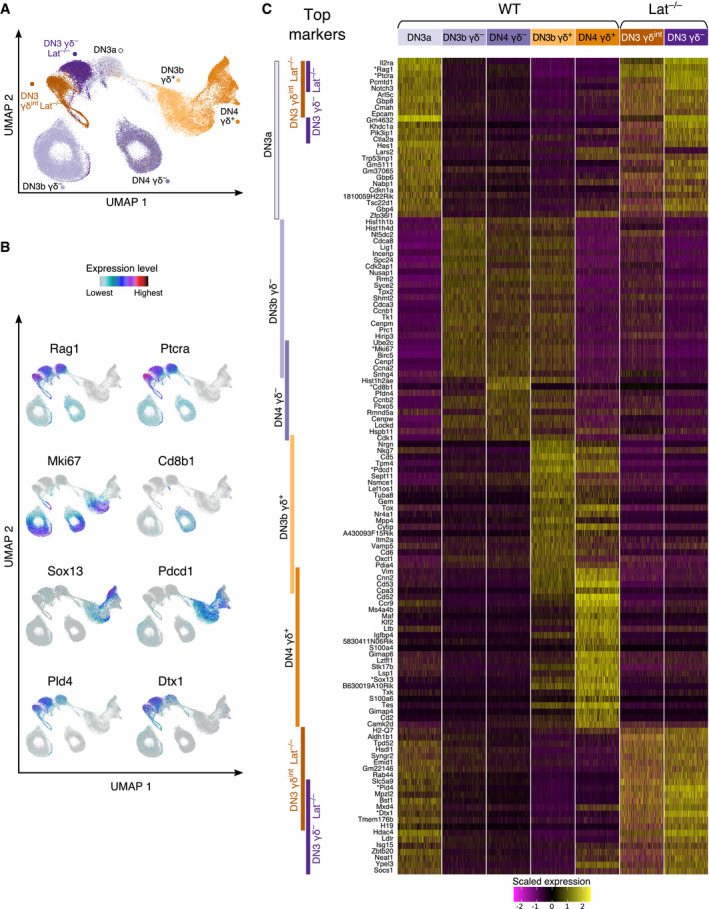
Single‐cell RNAseq analysis of DN3 γδ^−^ and DN3 γδ^int^ cells from *Lat* ^−/−^ thymus A, BUMAP plots of Dataset 3 which contains five WT (DN3a, DN3b γδ^+^, DN4 γδ^+^, DN3b γδ^−^, and DN4 γδ^−^) and two *Lat* ^−/−^ (DN3 γδ^−^ and DN3 γδ^int^) scRNAseq datasets. The UMAP plot is colored according to sorted samples (A), and to expression levels of the specified genes (B).CHeatmap showing scaled expression of top 25 DEG characterizing each of the seven sorted samples of Dataset 3. The names of the sorted samples are specified at the top of the heatmap, and 3,000 cells were randomly selected for each sample. Each column represents the expression profile of a single cell. The sample from which a DEG is derived is indicated by colored bars on the left. Bar superpositions specify DEG that are found in more than one sample. The genes displayed in panel B are highlighted with a star. Gene expression is color coded with a scale based on *z*‐score distribution, from −2 (purple) to 2 (yellow).

scRNAseq approaches offer the unprecedented possibility to analyze with single‐cell resolution the transcriptome of those rare LAT‐deficient DN3 γδ^int^ cells. More specifically, it offered the opportunity to determine whether the expression of a γδ TCR at their surface was associated with most hallmarks of γδ T‐cell lineage specification (Fig 4), or constituted their unique γδ T‐cell lineage marker. This last occurrence will formally demonstrate at single‐cell resolution that the signals delivered by the γδ TCR‐LAT axis have an instructive role in adult γδ T‐cell lineage commitment. Using a workflow similar to that developed for WT DN thymocytes (Fig 1A), the LAT‐deficient DN3 γδ^−^ and LAT‐deficient DN3 γδ^int^ cell subsets found in *Lat* ^−/−^ x *Trdc*‐*H2BEGFP* thymus were sorted and subjected to scRNAseq (Fig EV4B and C). After controlling the quality of our sequencing data (see Materials and Methods), the resulting LAT‐deficient DN3 γδ^−^ and LAT‐deficient DN3 γδ^int^ cell datasets comprised of 7704 and 5979 cells, respectively, that were merged with Dataset 1 to generate Dataset 3. The UMAP plot corresponding to Dataset 3 had the same overall shape as that obtained for Dataset 1 (Figs 2 and 5A). A γδ T‐cell and αβ T‐cell lineage branches emerged from DN3a cells and LAT‐deficient DN3 γδ^−^, and LAT‐deficient DN3 γδ^int^ cells were located on the αβ T‐cell lineage branch in close contiguity to WT DN3a cells (Fig 5A).

### Signaling‐proficient γδ TCR is required to commit DN3 γδ^int^ cells to the γδ T‐cell lineage

To confirm that the positions occupied by the LAT‐deficient DN3 γδ^−^ and LAT‐deficient DN3 γδ^int^ cells within the UMAP were consistent with their transcriptomics profile, we used the Seurat FindAllMarkers function to define marker genes for each of the seven sorted samples of Dataset 3 and filtered them to keep only the top 25 DEG (see Materials and Methods). As expected on the basis of former work (Mingueneau *et al*, 2013), the top DEG of WT DN3a cells comprised transcripts coding for components of the Notch signaling pathway (*Notch3*) and for the Notch targets *Hes1*, *Il2ra*, and *Ptcra* (Fig 5B and C). Most of them were downregulated in WT thymus at the DN3a → DN3b γδ^−^ and DN3a → DN3b γδ^+^ transitions. WT DN3a cells shared 9 of the top 25 DEG with LAT‐deficient DN3 γδ^−^ and LAT‐deficient DN3 γδ^int^ cells. The top DEG common to WT DN3a, LAT‐deficient DN3 γδ^−^ and LAT‐deficient DN3 γδ^int^ stages corresponded to *Ptcra*, *Rag1*, and *Notch3* and were all repressed in WT DN3b γδ^+^ cells.

**Figure 6:**
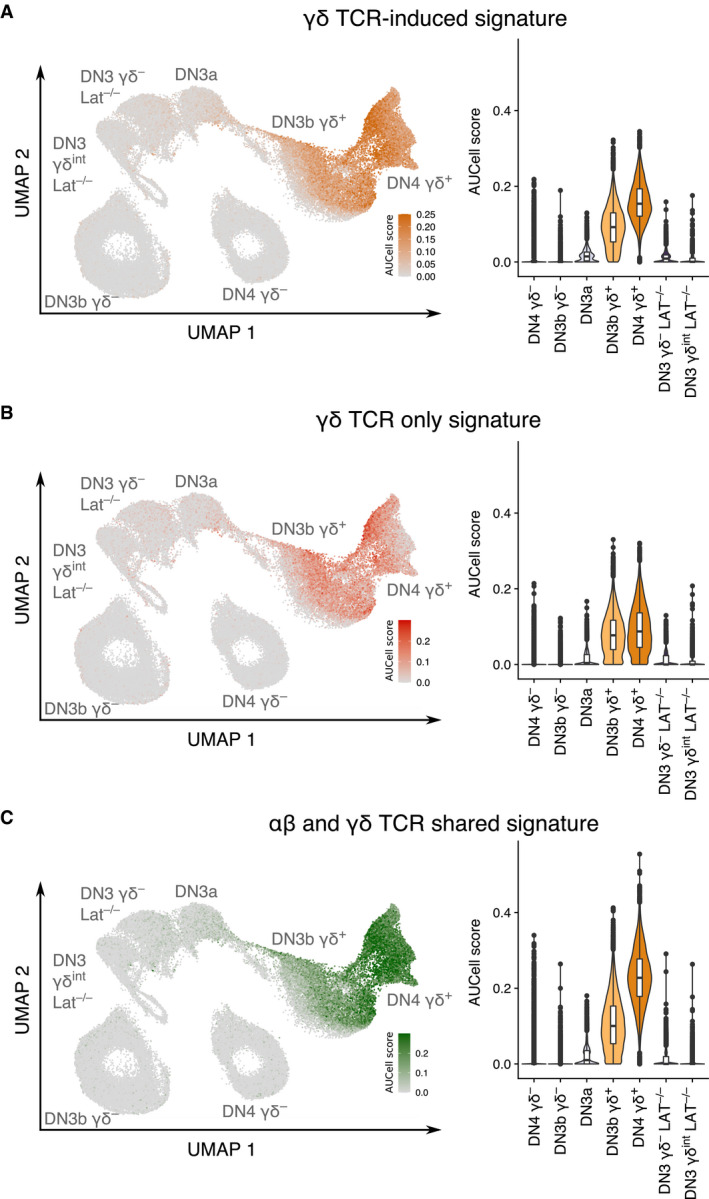
*Lat* ^−/−^ DN3 γδ^int^ T cells do not express the γδ lineage commitment signature A–CAUCell scores for the γδ TCR‐induced signature (A), γδ TCR‐only signature (B), and αβ and γδ TCR shared signature (C) are displayed on UMAP (left) and violin (right) plots of the seven sorted subsets corresponding to Dataset 3. As expected, AUCell scores for the three signatures were very low for DN3a cells, low for WT DN3b γδ^−^ and WT DN4 γδ^−^ cells, and high for WT DN3b γδ^+^ and WT DN4 γδ^+^ cells.

The top DEG of both WT DN3b γδ^+^ and WT DN3b γδ^−^ cells corresponded to genes linked to the cell cycle and to high metabolic activity. As expected, they were downregulated in WT DN4 γδ^+^ cells and remained expressed in WT DN4 γδ^−^ cells. Consistent with our previous result (Fig EV1), the sole αβ T‐cell lineage mark identified among the WT DN4 γδ^−^ top DEG corresponded to *Cd8b1*. In contrast, several genes among the top WT DN3b γδ^+^ and DN4 γδ^+^ DEG constituted hallmarks of the γδ T‐cell lineage (*Sox13*, *Maf*, and *Cpa3*), and those genes were absent or faintly expressed by LAT‐deficient DN3 γδ^int^ cells (Fig 5B and C). As a result, the LAT‐deficient DN3 γδ^int^ cells showed very low AUCell scores for the TCR γδ‐induced signature and its γδ TCR only and γδ and αβ TCR shared components (Fig 6A–C). Importantly, LAT‐deficient DN3 γδ^int^ cells did not express *Cd8b1*, the sole hallmark of early αβ T–cell specification (Fig 5B and C). Therefore, in the absence of a functional γδ TCR‐LAT signaling axis, no blatant mark of γδ T‐cell lineage specification can be found in DN3 γδ^int^ cells aside from γδ TCR expression. Signaling‐proficient γδ TCR is thus required to commit DN3 γδ^int^ cells to the γδ T‐cell lineage, formally demonstrating that our γδ TCR‐induced signature is indeed fully induced by the γδ TCR and does not contain components pre‐existing to γδ TCR induction.

### Revisiting the tightness of the developmental blockade occurring in *Lat* ^−/−^ thymus

**Figure 7:**
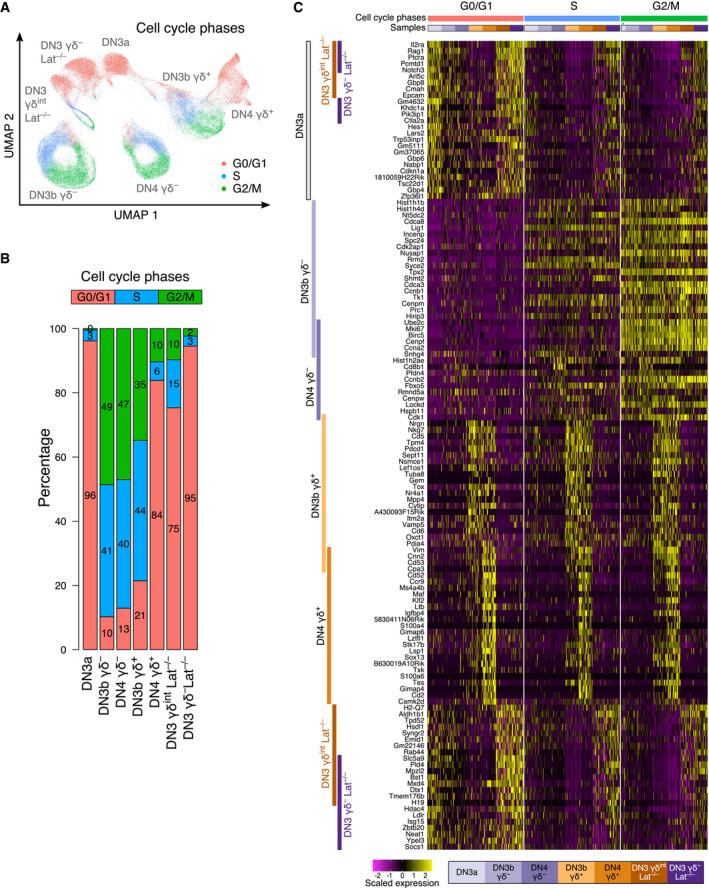
Impact of the cell cycle status on the scRNAseq expression profiles of DN3 γδ^−^ and DN3 γδ^int^ cells from *Lat* ^−/−^ thymus UMAP plots of sorted cell subsets from Dataset 3 colored according to cell cycle phases.Bar plot showing the percentage of cells in the G0/G1 (red bars), S (blue bars), and G2/M (green bars) phases of the cell cycle among each of the seven sorted cell subsets of Dataset 3.Heatmap showing scaled expression of up to 25 DEG from each of the seven sorted cell subsets of Dataset 3. In contrast to Fig 5C, the analyzed cells were further subdivided according to cell cycle phases (G0/G1, S, and G2/M) and 100 cells were randomly selected and analyzed per sample and per phase. The origin of each cell sample is indicated above columns and the color code corresponding to each seven sorted cell subsets is indicated at the bottom of the heatmap. A green star indicates that in the case of DN3a cells, only 32 cells were in G2/M phase and analyzed.

The lack of LAT is thought to induce a tight developmental blockade preventing DN3a cells to progress further along the αβ and γδ paths (Zhang *et al*, 1999; Nunez‐Cruz *et al*, 2003; Muro *et al*, 2015). Unexpectedly, single‐cell resolution analysis of the cycling status of the DN3 and DN4 subsets of Dataset 3 showed that approximately 25% of LAT‐deficient DN3 γδ^int^ cells were in S and G2M phases of the cell cycle as compared to 79% in WT DN3b γδ^+^ (Fig 7A and B). It suggested that in the absence of LAT, the γδ TCR expressed at the surface of DN3 γδ^int^ cells was still capable of delivering LAT‐independent signals to DN3 γδ^int^ cells leading a quarter of them to embark into the cell cycle. By revisiting the flow cytometry data corresponding to the DN3 cells found in *Lat* ^−/−^ thymus, we also found that approximately 30% of the LAT‐deficient DN3 γδ^int^ cells expressed CD71 (Appendix Fig S2), a hallmark of pre‐TCR and γδ TCR signaling (Fig 2D; Kelly *et al*, 2007). Moreover, minute numbers of LAT‐deficient DN4 γδ^int^ cells were also observed (Fig EV4A). Therefore, in contrast to former reports, the developmental blockade resulting from the lack of LAT is not as tight as previously described.

Our single‐cell resolution analysis offered us the unique opportunity to determine at the transcriptional level whether the cycling LAT‐deficient DN3 γδ^int^ cells that likely received some LAT‐independent γδ TCR signals differed from the non‐cycling LAT‐deficient DN3 γδ^int^ cells and showed some hints of γδ T‐cell specification. Accordingly, we reorganized the heatmap corresponding to the seven samples of Dataset 3 based on their cell cycling status (G0/G1, S, or G2/M; Fig 7C). It confirmed that most of the WT DN3b γδ^−^/DN4 γδ^−^ cells and WT DN3b γδ^+^ top DEG were characteristic of highly cycling cells (*Mki67*, *Ccnb1*, *Ccnb2*, *Nusap1*, *Rrm2*, *Cdca3*, *Birc5, Ube2c*, *Cenpm*, *Prc1*, *Cenpf*, *Ccna2*, *Hist1h1b*, *Hist1h4d*, *Hist1h2ae*, *Pfdn4, Fbxo5*, *Rmnd5a*, *Cenpw*, *and Ckk1*), and were switched off in the G0/G1 cells present in the seven analyzed cell subsets. The WT DN3a top DEG were repressed during the transition to WT DN3b γδ^−^ and WT DN3b γδ^+^ cells. The LAT‐deficient DN3 γδ^int^ cells that were in S and G2/M also repressed the WT DN3a DEG but to a smaller extent than WT DN3b γδ^−^ and DN3b γδ^+^ cells. Most remarkably, none of the WT DN3b γδ^+^ and DN4 γδ^+^ top DEG, which primarily corresponded to genes involved in γδ T‐cell differentiation and function, was induced in LAT‐deficient DN3 γδ^int^ cells, including those that managed to enter the cell cycle. Therefore, even the LAT‐deficient DN3 γδ^int^ cells that started cycling via LAT‐independent γδ TCR signals were unable to turn on the program associated with γδ T‐cell lineage commitment. Accordingly, our data formally demonstrate at single‐cell resolution that the γδ TCR‐LAT signaling axis exerts a key inductive role in adult γδ T‐cell lineage commitment and specification.

## Discussion

We investigated at single‐cell resolution the developmental bifurcation leading to αβ and γδ T‐cell lineage specification in the thymus of adult WT mice and mice deprived of the LAT adaptor. It offered the unique possibility to compare the transcriptome of DN3a and γδ‐selected WT DN3b γδ^+^ cells to that of DN3 cells that succeeded in expressing a γδ TCR but failed delivering LAT‐dependent signals. It demonstrated at single‐cell resolution that γδ TCR signals build upon a transcriptomic cell state that shows no sign of γδ T‐cell lineage commitment, and thus that signaling‐proficient γδ TCR is required to commit DN3 cells to the γδ T‐cell lineage. We also defined at single‐cell level, the commonalities and differences in the transcriptional signatures resulting from pre‐TCR, γδ TCR, and αβ TCR signaling at distinct stages of adult intrathymic development. Genes encoding several markers of signal strength (*Cd69*, *Cd5,* and *Nr4a1*) were induced by the pre‐TCR, the γδ TCR, and the αβ TCR in DN3b γδ^+^, DN3b γδ^−^/DN4 γδ^−^, and DP cells, respectively. Consistent with the signal strength model of αβ versus γδ T‐cell lineage commitment, the magnitude of their induction was lower during β selection as compared to γδ selection, as well as to αβ selection (Figs 4 and EV3). Moreover, the transcriptional signature induced by the γδ TCR at the DN3a → DN3b γδ^+^ transition was more similar to that induced by the αβ TCR at the DP_small_ → DP_CD69+_/SP transition than to the pre‐TCR signature. Consistent with previous reports (Mingueneau *et al*, 2013; Taniuchi, 2018; Rothenberg, 2019), conventional DP_CD69+_/SP cells differed, however, from DN4 γδ^+^ cells in that they postponed most of their functional specialization after they leave the thymus and receive cytokine signals from antigen‐laden dendritic cells in the periphery.

Both the DN3b γδ^+^ cells and DN3b γδ^−^/DN4 γδ^−^ cells constitute “transit‐amplifying cells” that after dividing rapidly progress to differentiate into mature γδ and αβ T cells, respectively. To obtain the same fine‐grained picture of these “transit‐amplifying cells,” we subjected comparable numbers of sorted DN3b γδ^+^, DN3b γδ^−^, and DN4 γδ^−^ cells to scRNAseq. However, it is important to emphasize that the DN3b γδ^+^ and DN3b γδ^−^ /DN4 γδ^−^ cells correspond to 0.4% and 25%, respectively, of adult DN cells. Therefore, the presence in an adult thymus of approximately 300‐fold more DP cells (the direct progeny of DN4 γδ^−^ cells) than DN4 γδ^+^ cells (the direct progeny of DN3b γδ^+^ cells) is commensurate to the sizes of the DN3b γδ^+^ and DN3b γδ^−^/DN4 γδ^−^ “transit‐amplifying cell” compartments. These distinct sizes might result from the distinct mitogenic capacity of the pre‐TCR and γδ TCR, and/or from the lower occurrence among DN3a cells of coincident productive *Trg* and *Trd* gene rearrangements as compared to that of productive *Trb* gene rearrangements.

Our single‐cell resolution analysis also demonstrated that the heterogeneity present among DN3b γδ^+^, DN3b γδ^−^, and DN4 γδ^−^ cells was primarily due to their distinct cycling status, whereas that found in DN4 γδ^+^ cells reflected their diversification into distinct γδ T‐cell effector subsets. In contrast, DN3a cells were more homogeneous, suggesting that after assembling in DN3a cells, the pre‐TCR and γδ TCR use the same signal transduction machinery. Therefore, the different developmental outcomes resulting from the firing of the pre‐TCR and γδ TCR have to result from their distinct subunit architectures or ligand dependence (Dutta *et al*, 2021). In addition, the pre‐TCR and γδ TCR do not work in isolation and other receptors contribute to enforce αβ and γδ T‐cell lineage specification (Dutta *et al*, 2021). Likewise, the effector programming of DN4 γδ^+^ cells subsets also depends on environmental cues, such as cytokines, and costimulatory molecules (Fiala *et al*, 2020; Chen *et al*, 2021).

In mature αβ T cells, TCR‐triggered signals divide into multiple branches at the level of the plasma membrane, resulting in the formation of multiple signalosomes nucleating around transmembrane receptors or adaptors such as LAT, CD6, CD5, and LAX1 (Mori *et al*, 2021). They contribute to TCR signal diversification and to the initiation of negative feedback loops. Accordingly, in the absence of LAT, TCR engagement in mature αβ T cells still induce a partial signaling cascade that prevents the differentiation of effector T cells (Mingueneau *et al*, 2009). Akin to LAT‐deficient mature αβ T cells, cross‐linkage of the γδ TCR expressed on LAT‐deficient DN3 γδ^int^ cells failed to induce ERK phosphorylation but triggered normal levels of AKT phosphorylation (Muro *et al*, 2018). Therefore, the partial LAT‐independent γδ TCR signals induced in LAT‐deficient DN3 γδ^int^ cells likely account for the entry into the cell cycle of 0.3% of them and in turn to their aborted progression beyond the DN4 stage. Although our study provides an unprecedented single‐cell resolution picture of the role of γδ TCR signaling in the specification of the adult γδ‐T cell lineage, one of its limitations must be noted. It results from the extremely low surface expression of the pre‐TCR on DN3 cells as compared to that of the γδ TCR, a feature precluding identification of pre‐TCR^+^ DN3 cells in WT and LAT‐deficient mice and in turn the definition of their single‐cell transcriptome.

In conclusion, our study demonstrated that γδ TCR signals build upon a γδ T‐cell uncommitted transcriptomic state to initiate γδ T‐cell lineage specification in DN3 cells of adult thymus, a result supporting the instructive model of γδ T‐cell lineage commitment. In addition, the single‐cell resolution afforded by scRNAseq provided a more complete view of the first checkpoint encountered by developing T cells and of the extent of its perturbation in the absence of the LAT adaptor. Charting the distinct signal transduction networks at the basis of successful β selection and γδ selection remains an important challenge owing to the small numbers of early developing T cells in which those events occur. Most single‐cell studies focus on nucleic acids and, as long as specific antibodies are available, on proteins and post‐translational modifications. However, protein complexes, rather than individual protein, are the functional units of signaling pathways, suggesting that new advances in single‐cell proteomics and interactomics by mass spectrometry will help further comparison of the β‐selection and γδ‐selection checkpoints.

## Materials and Methods

### Mice

Mice were bred under specific pathogen‐free conditions in CIPHE animal facilities and handled in accordance with Institutional Committee and European guidelines for animal care. C57BL/6 mice were purchased from Janvier Labs and *Lat* ^−/−^ x *Trdc*‐*H2BEGFP* mice have been described (Prinz *et al*, 2006).

### Cell preparation

Cells from thymus and spleen were extracted as described in the IMPRESS protocol (https://www.mousephenotype.org/impress/ProcedureInfo?procID=201). Briefly, organs were disrupted using OctoGentleMACS system (Miltenyi Biotec) with 35 mg collagenase II (Serlabo) and 50 μg DNAse I (Sigma) for 20 min at room temperature (RT). Enzymatic reaction was stopped with EDTA (10mM, Invitrogen). Red blood cells present in spleens were lysed for 1 min at RT using an ammonium–chloride–potassium (ACK) lysis solution (eBioscience). Cell suspensions were filtered with 70μm cell strainers (BD Falcon) and counted using Sytox Green or Sytox Red (Invitrogen) to exclude dead cells.

### Depletion of CD4^+^, CD8^+^, and CD4^+^CD8^+^ cells from thymus

Cells extracted from pooled thymus were depleted of CD4^+^, CD8^+^, and CD4^+^CD8^+^ T cells using Dynabeads Mouse CD4 and Dynabeads Mouse CD8 according to the manufacturer’s protocol (Invitrogen Life Technologies). Proper depletion was validated by staining the resulting cell suspension with anti‐CD4‐BV421 (RM4‐5), anti‐CD8α‐PE (53‐7.3), and anti‐CD8β‐PE (H35‐17.2) antibodies from BD Biosciences. The remaining DN‐enriched thymocytes were stained with dead cell marker Sytox Green (Invitrogen) and then live cells were counted on an Attune NXT system (Life Technologies).

### Flow cytometry analysis

Samples were first stained with Fixable Viability Stain 440 UV (BD Biosciences), and incubated for 15 min on ice with anti‐CD16/32 antibody (clone 2.4G2) to block Fc receptors. Samples were then stained with the following antibodies CD3ε‐BB700 (145‐2C11), CD4‐BUV737 (RM4‐5), CD8α‐BV650 (53‐6.7), CD25‐BUV395 (PC61), CD27‐PE (LG.3A10), CD69‐BV605 (H1.2F3), CD117‐PE‐CF594 (2B8), and CD161‐APC‐Cy7 (PK136), all from BD Biosciences, with CD5‐PE‐Cy7 (53‐7.3), CD24‐A700 (M1/69), CD44‐APC (IM7), and CD71‐BV421 (RI7217), all from Biolegend, and with TCRδ‐PE‐Cy5 (GL‐3) from Invitrogen. Multiparameter FACS acquisition was performed on a Fortessa LSRII SORP system (BD Biosciences) and data analyzed with FACSDiva 9.0 (BD Biosciences) and FlowJo 10.7.1 (BD Biosciences) software. Doublets were systematically excluded based on side scatter (SSC) and forward scatter (FSC) parameters.

### Antibodies

Antibodies used for flow cytometry are listed in Appendix Table S1.

### Preparation of single‐cell RNA‐sequencing libraries

#### DN3‐DN4 cell libraries generation

Total *Lat* ^−/−^ x *Trdc*‐*H2BEGFP* thymocytes and DN‐enriched WT thymocytes were labeled with CD3ε‐BB700 (145‐2C11), CD4‐BUV737, CD8α‐APC (53‐6.7), CD25‐BUV395, CD44‐PE, CD69‐BV605, CD117‐PE‐CF594, and CD161‐BV650, all from BD Biosciences; CD5‐PE‐Cy7, CD24‐A700, and CD71‐BV421, all from Biolegend; and TCRδ‐PE‐Cy5 (Invitrogen). For both types of samples, DAPI (Invitrogen) was used as a viability dye. DN thymocyte subsets were sorted as specified in Figs 1A and EV4B using a BD FACSAria SORP (BD Biosciences). Doublets were systematically excluded based on side scatter (SSC) and forward scatter (FSC) parameters. Mouse splenic B cells (CD19^+^MHC class II^+^) were sorted after labeling with CD19 (1D3) and IA‐IE (M5/114.15.2) both from BD Biosciences, and were used as internal standard for generating Datasets 1 and 3. After sorting, cells were washed in PBS (Sigma‐Aldrich), supplemented with 0.04% BSA (Sigma‐Aldrich), and counted using Attune NXT system (Thermo Fischer Scientific). A total of 18,000 sorted cells corresponding to each of the five DN thymocyte subsets of interest were spiked with 500 sorted splenic B cells, and then loaded on a Chromium Chip B (one cell‐subset per well), and droplet encapsulated with a Chromium Controller (10X Genomics). Single‐cell cDNA synthesis and sequencing libraries were prepared with Chromium Single Cell 3’ v3 Library and Gel Bead kit (10X Genomics) according to manufacturer’s instructions.

#### DN‐enriched and total WT thymocyte libraries generation

DN‐enriched and total WT thymocyte suspensions were sorted based on viability using DAPI (Invitrogen) as specified in Fig EV2A using a BD FACSAria SORP (BD Biosciences). Doublets were systematically excluded based on SSC and FSC parameters. Thawed human PBMC (huPBMC) cells were counted using Sytox Green (Invitrogen), stained with DAPI (Invitrogen), sorted for live cells, and were used as an internal standard for generation of Dataset 2. After sorting, cells were washed and counted as described above. A total of 18,000 sorted cells corresponding either to live DN thymocytes or to live total thymocytes were spiked with 5,000 sorted live huPBMC, then loaded on a Chromium Chip B (one cell‐subset per well) and droplet‐encapsulated with a Chromium Controller (10X Genomics). Single‐cell cDNA synthesis and sequencing libraries were prepared as described above.

### Sequencing and data analysis

Sequencing was performed by Macrogen (Seoul, South Korea) on an Illumina HiSeq X platform and the following parameters were applied: Read 1:26 cycles; i7:8 cycles; and Read 2:57 cycles. mRNA libraries were preprocessed using CellRanger count (version 5.0.1).

### Generation of datasets 1 and 3

#### Dataset 1

Sequencing reads obtained from DN3‐DN4 subsets sorted from WT and *Lat* ^−/−^ x *Trdc*‐*H2BEGFP* thymus were aligned to a modified version of the mm10 mouse genome containing the *Trdc*‐*H2BEGFP* edited version of the *Trdc* gene and quantified. The mRNA data matrices were imported into R (v3.6.3) and downstream analysis was performed with the Seurat package (version 3.1.5) (Butler *et al*, 2018; Stuart *et al*, 2019). Low‐quality cells corresponding to cells expressing < 200 genes or > 15% of mitochondrial‐associated genes were filtered out and genes expressed in less than three cells were removed. The five WT sorted samples (DN3a, DN3b γδ^−^, DN4 γδ^−^, DN3b γδ^+^, and DN4 γδ^+^) were first merged with the Seurat merge function. Before mRNA expression analysis, *Trdc*‐*H2BEGFP* and *Trdc* gene counts were summed up in a single feature called “Trdc.” Then, expression raw counts were normalized and scaled using, respectively, the Seurat NormalizeData and ScaleData functions. The top 2,000 variable genes were selected (FindVariableFeatures function; selection method = “vst”) to perform a dimensionality reduction using the principal component analysis method (RunPCA function), and a UMAP was computed using the 20 first components (RunUMAP function). Cell clusters were identified using Seurat FindClusters function with Louvain Algorithm option (using 20 dimensions and a resolution of 1). We filtered out unwanted cell clusters (non‐T cells, B‐cell/T‐cell doublets, and low‐quality cells having a low percentage of ribosomal genes). Then, to obtain Dataset 1, we reselected the most 2,000 variable genes and performed a second UMAP dimensionality reduction (with RunPCA and RunUMAP functions using 30 dimensions and k.param option 50) and clustering with a resolution of 0.4. To analyze γδ lineage diversification, we selected cells corresponding DN3a to DN4 γδ^+^ T cells and switched to a finer clustering resolution (1.5).

#### Dataset 3

To generate Dataset 3, we added the *Lat* ^−/−^ x *Trdc*‐*H2BEGFP* sorted samples (*Lat* ^−/−^ DN3 γδ^−^ and *Lat* ^−/−^ DN3 γδ^int^) to the five sorted WT samples (DN3a, DN3b γδ^−^, DN4 γδ^−^, DN3b γδ^+^, and DN4 γδ^+^) using the Seurat merge function and proceeded as described previously for Dataset 1. Considering that the spike‐in B cells from the *Lat* ^−/−^ DN3 γδ^int^ were not properly superimposed because of a different proportion of ribosomal mRNA, we regressed out the proportion of ribosomal mRNA using the Seurat scaledata function. To obtain a UMAP plot where B cells were properly superimposed, we recalculated the UMAP coordinates and clustering (30 dimensions and resolution 1). To obtain Dataset 3, we filtered out unwanted clusters and reselected the 2,000 most variable genes to perform UMAP dimensionality reduction (using 50 dimensions and k.param option 50) and clustering with a resolution of 0.4 corresponding to a merged and cleaned Dataset 3.

### Generation of dataset 2

Using CellRanger, sequencing reads from whole WT thymus and from WT DN‐enriched samples were aligned to the GRCh38/mm10 mouse and human composite genome and gene count were quantified. Gene expression matrix was slimmed down to keep only mm10 features using CollapseSpeciesExpressionMatrix function and human/mouse cell doublets were removed. Low‐quality cells were also filtered out (i.e., cells expressing < 200 genes or > 10% of mitochondrial‐associated genes) and genes expressed in less than three cells were removed. Expression raw counts were then normalized using Seurat NormalizeData function and samples were merged with Seurat merge function. The top 2,000 variable genes were selected (FindVariableFeatures function; selection method = “vst”) to perform a dimensionality reduction using the principal component analysis method (RunPCA function). A UMAP was computed using the 20 first components (RunUMAP function) and cells were clustered with the FindNeighbors (20 dimensions) and FindClusters (0.6 resolution) functions. Non‐T‐cell clusters were filtered out and the cell cycle variation effects were regressed out using the ScaleData function. To obtain a merged and cleaned Dataset 2, the top 2,000 variable genes were reselected to perform a second UMAP dimensionality reduction (using 30 dimensions) and clustering with a resolution of 0.42.

### Gene expression analysis and statistical analysis

DEG were identified using the FindAllMarkers Seurat function with the default parameters (Wilcoxon rank sum test for genes with a minimum 0.25 log fold change between the two groups of cells and expressed in at least 10% of cells in either of the two populations tested). DEG between two samples were identified using the FindMarkers function using the same parameters as the FindAllMarkers Seurat function. For the γδ TCR‐induced signature, pre‐TCR‐induced signature, and top DEG markers of Dataset 3, we added a filter to keep only genes having an adjusted *P*‐value of 0 and expressed in < 25% of the cells of the compared samples group (pct.2 < 25%). A *P*‐value of 0 corresponds to *P*‐value < 2.225074e^−308^ due to R limitations. Cell cycle phases were scored used the CellCycleScoring Seurat function. To identify cell samples in which a given signature is active, we assigned a signature score to each cell sample using AUCell.

## Author contributions

**Bernard Malissen:** Conceptualization; Resources; Formal analysis; Supervision; Funding acquisition; Validation; Investigation; Methodology; Writing—original draft; Project administration; Writing—review & editing. **Sara Scaramuzzino:** Formal analysis; Validation; Investigation; Visualization; Methodology. **Delphine Potier:** Conceptualization; Data curation; Software; Formal analysis; Supervision; Validation; Investigation; Visualization; Methodology. **Robin Ordioni:** Investigation; Methodology. **Pierre Grenot:** Investigation; Methodology. **Dominique Payet‐Bornet:** Data curation; Software; Formal analysis; Supervision; Validation; Investigation; Visualization; Methodology; Writing—original draft. **Hervé Luche:** Conceptualization; Supervision; Validation; Investigation; Methodology.

In addition to the CRediT author contributions listed above, the contributions in detail are:

BM and HL conceived the project, SS, PG, and RO generated the data, SS, DP‐B, and DP analyzed the data and prepared the figures, and BM wrote the manuscript with contributions from all authors.

## Supporting information



Appendix

Expanded View Figures PDF

## Data Availability

The transcriptomics data have been deposited in the Gene Expression Omnibus public database under accession number GSE184545 (http://www.ncbi.nlm.nih.gov/geo/query/acc.cgi?acc=GSE184545). GSE184545 comprises of two sub‐series denoted as GSE184483 (http://www.ncbi.nlm.nih.gov/geo/query/acc.cgi?acc=GSE184483) and GSE184544 (http://www.ncbi.nlm.nih.gov/geo/query/acc.cgi?acc=GSE184544) corresponding to Datasets 1 and 3 and to Dataset 2, respectively.
