# Supplementary material for: Single‐cell transcriptomics uncovers an instructive T‐cell receptor role in adult γδ T‐cell lineage commitment
Source: EMBO J. 2022 Feb 7;41(5):e110023. doi: 10.15252/embj.2021110023 (PMC8886544; doi:10.15252/embj.2021110023)
Supplement: Supplementary file 1 — Appendix [file EMBJ-41-e110023-s003.pdf]

Appendix

Appendix Figure S1. Distribution of the cells present in each of the 11 cell clusters identified in DN3a, DN3b $\gamma\delta^+$ and DN4 $\gamma\delta^+$ cells according to the G0/G1, S and G2/M phases of the cell cycle

Appendix Figure S2: Expression of CD71 on *Lat*$^{-/-}$ DN3 $\gamma\delta^{int}$ cells.

Appendix Table S1: Table of antibodies used for flow cytometry analysis and FACS sorting.

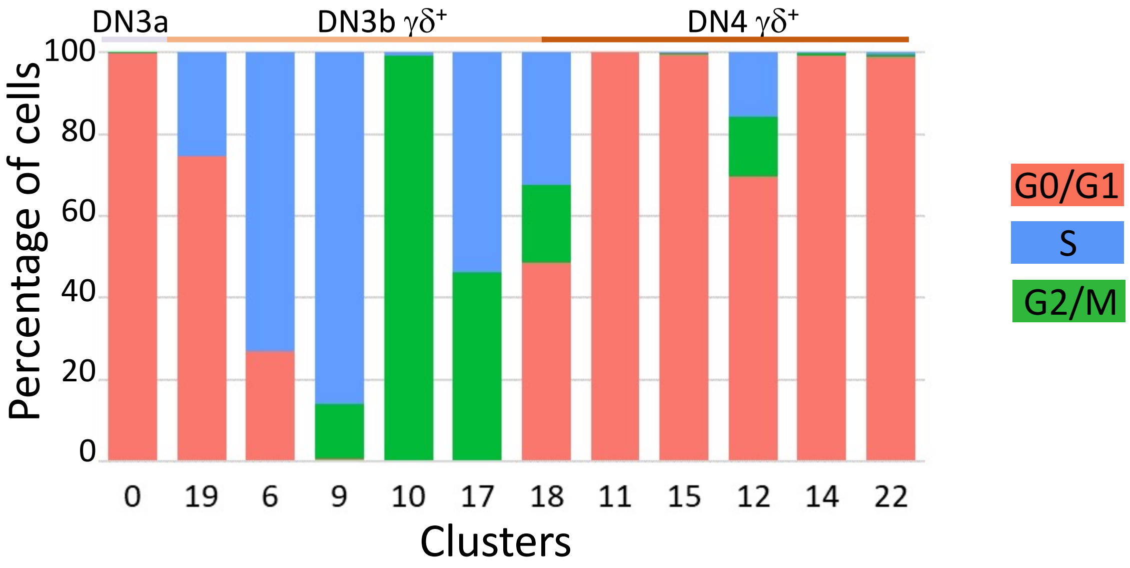

Appendix Figure S1. Distribution among the G0/G1, S and G2/M phases of the cell cycle of the cells present in each of the 11 cell clusters identified in DN3a, DN3b γδ$^+$ and DN4 γδ$^+$ cells. The bar plot shows for each of the 11 cells clusters identified among DN3a, DN3b γδ$^+$ and DN4 γδ$^+$ cells (see Fig 3A) the percentage of cells found in the G0/G1, S and G2/M phases of the cell cycle

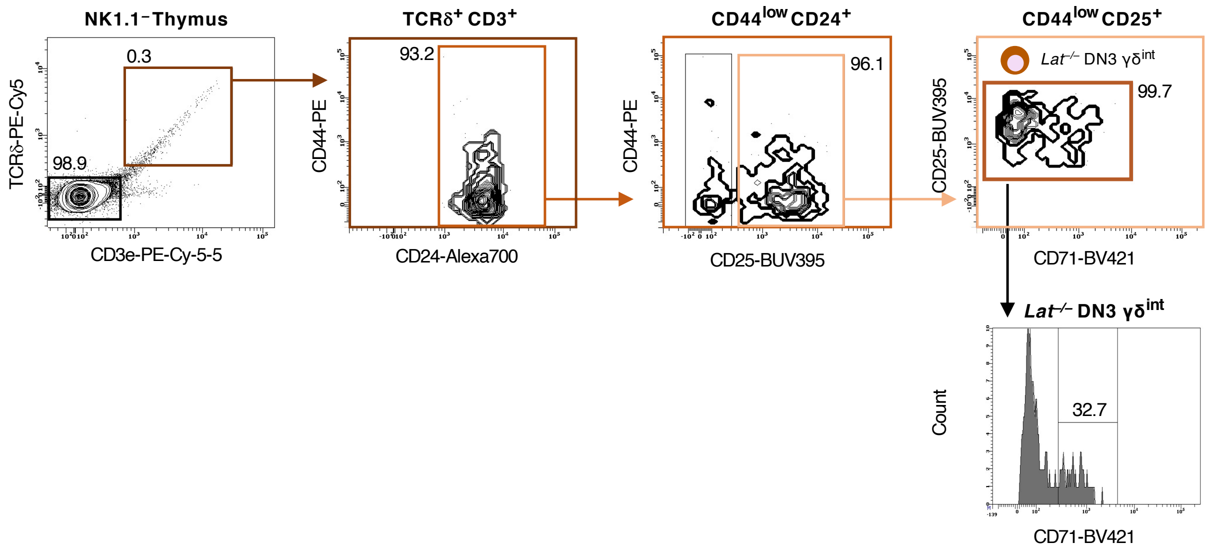

Appendix Figure S2 : Expression of CD71 on *Lat−/−* DN3 γδ^{int} cells. FACS plots showing the gating strategy used to sort DN3 γδ^{int} populations from *Lat−/−*-TCRd-H2BEGFP mice. A histogram shows the expression of CD71 on the sorted subset DN3 γδ^{int} *Lat−/−*.

Appendix Table S1 : Table of antibodies used for flow cytometry analysis and FACS sorting.

Antigen	Fluorochrome	Clone	Company	Cat Number	RRID	Immunophenotyping	Cell Sorting : Thymic cells	Cell Sorting : B cells	Depletion validation
CD3e	BB700	145-2C11	BD Biosciences	745836	AB_2743282	✓	✓	✗	✗
CD4	BUV737	RM4-5	BD Biosciences	564933	AB_2732918	✓	✓	✗	✗
CD4	BV421	RM4-5	BD Biosciences	740007	AB_2739779	✗	✗	✗	✓
CD5	PE-Cy7	53-7.3	Biologend	100622	AB_2562773	✓	✓	✗	✗
CD8a	APC	53-6.7	BD Biosciences	553035	AB_398527	✗	✓	✗	✗
CD8a	BV650	53-6.7	BD Biosciences	563234	AB_2738084	✓	✗	✗	✗
CD8a	PE	53-7.3	BD Biosciences	561095	AB_2034011	✗	✗	✗	✓
CD8b	PE	H35-17.2	BD Biosciences	550798	AB_393887	✗	✗	✗	✓
CD19	APC-Cy7	1D3	BD Biosciences	557655	AB_396770	✗	✗	✓	✗
CD24	A700	M1/69	Biologend	564237	AB_2738691	✓	✓	✗	✗
CD25	BUV395	PC61	BD Biosciences	564022	AB_2722574	✓	✓	✗	✗
CD27	PE-Cy7	LG.3A10	BD Biosciences	558754	AB_397106	✓	✗	✗	✗
CD44	APC	IM7	Biologend	103012	AB_312963	✓	✗	✗	✗
CD44	PE	IM7	BD Biosciences	553134	AB_394649	✗	✓	✗	✗
CD69	BV605	H1.2F3	BD Biosciences	563290	AB_2738120	✓	✓	✗	✗
CD71	BV421	RI7217	Biologend	113813	AB_10899739	✓	✓	✗	✗
CD117	PE-CF594	2B8	BD Biosciences	562417	AB_11154233	✓	✓	✗	✗
CD161	BV650	PK136	BD Biosciences	564143	AB_2738617	✗	✓	✗	✗
CD161	APC-Cy7	PK136	BD Biosciences	560618	AB_1727569	✓	✗	✗	✗
IA-IE	V500	M5/114.15.2	BD Biosciences	562366	AB_11153488	✗	✗	✓	✗
TCRd	PE-Cy5	GL-3	Invitrogen	15-5711-82	AB_468804	✓	✓	✗	✗

✓ Antiboby used in the experiment

✗ Antiboby not used in the experiment
