# Supplementary material for: Single‐cell transcriptomics uncovers an instructive T‐cell receptor role in adult γδ T‐cell lineage commitment
Source: EMBO J. 2022 Feb 7;41(5):e110023. doi: 10.15252/embj.2021110023 (PMC8886544; doi:10.15252/embj.2021110023)
Supplement: Supplementary file 2 — Expanded View Figures PDF [file EMBJ-41-e110023-s002.pdf]

Expanded View Figures

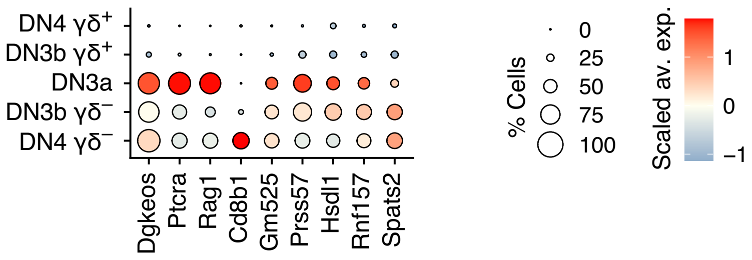

Figure EV1. Transcriptional footprint of pre-TCR selection.

Dot plot showing the expression level of genes characterizing the "pre-TCR-induced" signature among the DN3a, DN3b $\gamma\delta^{+}$, DN4 $\gamma\delta^{+}$, DN3b $\gamma\delta^{-}$, and DN4 $\gamma\delta^{-}$ sorted subsets of Dataset 1. The "pre-TCR-induced" signature was obtained by analyzing the genes differentially expressed between the DN3b $\gamma\delta^{-}$/DN4 $\gamma\delta^{-}$ and DN3b $\gamma\delta^{+}$ sorted samples and by selecting the genes upregulated in DN3b $\gamma\delta^{-}$/DN4 $\gamma\delta^{-}$ subset (adjusted *P*-value = 0 and expressed in < 25% of the DN3b $\gamma\delta^{+}$ cells). A fully identical gene list was generated when a *P*-value < 0.01 was used.

Figure EV2. Expression of the γδ TCR-induced signature across the whole sequence of intrathymic T-cell development.

A Schematic representation of the workflow used for scRNAseq analysis of total thymocytes and DN-enriched thymocytes and the generation of Dataset 2.

B UMAP plots of the cells corresponding to Dataset 2 and colored according to the defined clusters.

C Dot plot showing the expression level of selected genes among the eight cell clusters identified in Dataset 2. Dot color represents the scaled average expression of the gene of interest across the various clusters, whereas dot size indicates the percentage of cells expressing the specified gene.

D Violin plots showing AUCell scores of the γδ TCR-induced (top), γδ TCR-only signature (middle), and the "αβ and γδ TCR shared" (bottom) signatures among the specified subsets.

© 2022 The Authors

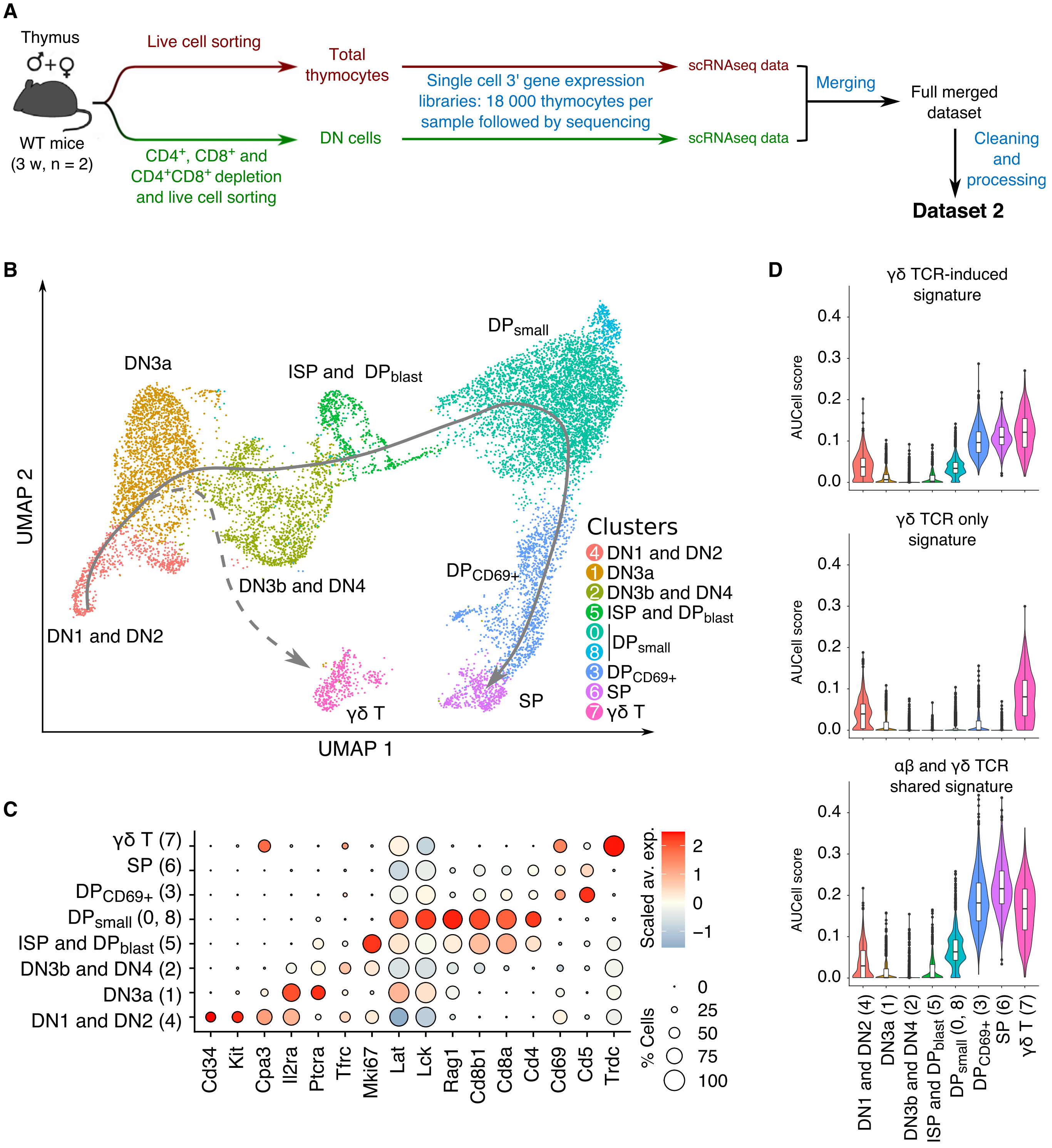

Figure EV2.

 © 2022 The Authors

Figure EV3. Analysis of the genes that are differentially expressed during the $DP_{small} \rightarrow DP_{CD69+}$/SP transition.

A Dot plot showing the expression level of the top genes which are upregulated during the $DP_{small} \rightarrow DP_{CD69+}$/SP transition among the eight cell clusters identified in Dataset 2 (genes shown are expressed in < 25% of DP_{small} cell, have a *P*-value = 0 and a log fold change > 0). Dot color represents the scaled average expression of the specified genes across the eight cell clusters, whereas dot size indicates the percentage of cells expressing the specified gene.

B The table summarized the results of differential gene expression analysis of $DP_{small} \rightarrow DP_{CD69+}$/SP transition using a Wilcoxon rank sum test (Seurat FindMarkers function used with default parameters). Only the genes corresponding to the γδ TCR-induced signature are shown. The average log fold change (FC) corresponds to the log fold change of the average expression between the two groups. Positive values indicate that the gene is more highly expressed in the DP_{CD69+}/SP group. The pct.1 DP_{CD69+}/SP and pct.2 DP_{small} values correspond to the percentage of DP_{CD69+}/SP and DP_{small} cells where the gene is detected, respectively. Adjusted *P*-value (p_val_adj) is based on Bonferroni correction using all genes in the dataset. The genes not differentially expressed are denoted as "NA" (not available). Accordingly, the γδ TCR-induced signature can be divided into two gene sets: gene set #1 consists of genes that are not upregulated in DP_{CD69+}/SP cells and gene set #2 consists of genes upregulated in DP_{CD69+}/SP cells. *P*-values are indicated for both gene sets. Therefore, gene set #1 was defined as the "γδ TCR only" signature and gene set #2 as the "αβ and γδ TCR shared" signature.

© 2022 The Authors

A

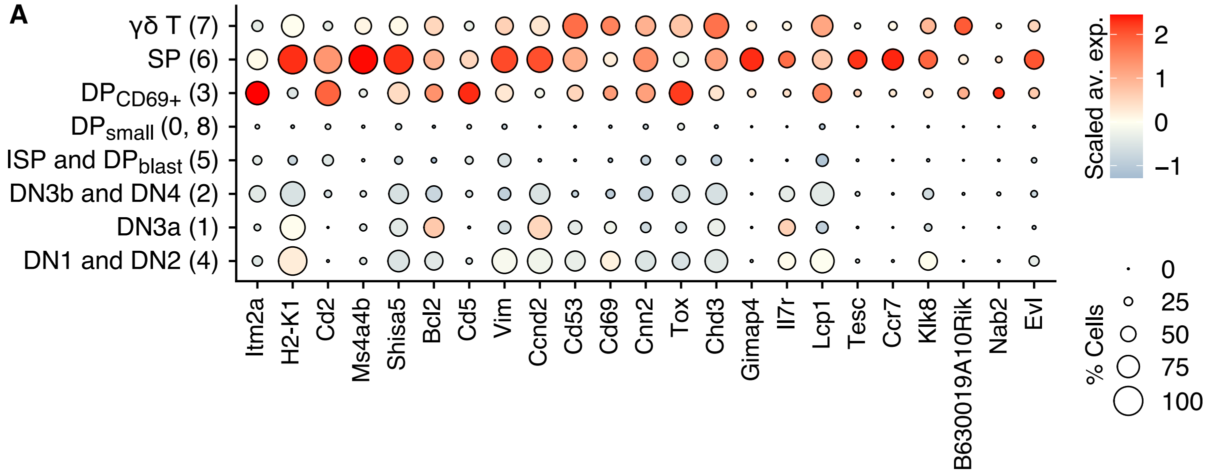

B

Genes of γδ TCR-induced signature	p_val	avg_logFC	Pct.1 'DP$_{CD69+}$/SP'	Pct.2 'DP$_{small}$'	p_val_adj
Sox13	NA	NA	NA	NA	NA
Etv5	NA	NA	NA	NA	NA
Blk	NA	NA	NA	NA	NA
Tuba8	NA	NA	NA	NA	NA
Tox2	NA	NA	NA	NA	NA
Gem	NA	NA	NA	NA	NA
Mpp4	NA	NA	NA	NA	NA
Nrgn	NA	NA	NA	NA	NA
Scin	NA	NA	NA	NA	NA
BC035044	NA	NA	NA	NA	NA
Bex6	NA	NA	NA	NA	NA
Rbpms	NA	NA	NA	NA	NA
Cpa3	NA	NA	NA	NA	NA
Coro2a	NA	NA	NA	NA	NA
Cd160	NA	NA	NA	NA	NA
Ccl4	NA	NA	NA	NA	NA
Gstp1	NA	NA	NA	NA	NA
Basp1	NA	NA	NA	NA	NA
Ppp1r16b	NA	NA	NA	NA	NA
Igfbp4	5.04e-152	0.649	0.159	0.008	9.45e-148
Trac	1.59e-185	0.578	0.952	0.629	2.98e-181
Cd52	3.99e-186	0.727	0.953	0.855	7.48e-182
Cd53	0.00e+00	1.151	0.594	0.032	0.00e+00
Cd5	0.00e+00	1.373	0.686	0.103	0.00e+00
Cd6	3.09e-161	0.621	0.525	0.181	5.78e-157
Arap2	3.68e-64	0.345	0.481	0.240	6.90e-60
Egr1	2.94e-202	0.771	0.262	0.027	5.51e-198
Nab2	0.00e+00	0.686	0.292	0.006	0.00e+00
Nr4a1	1.35e-140	0.682	0.168	0.014	2.53e-136
Pdcd1	5.93e-120	0.365	0.112	0.003	1.11e-115
St6gal1	2.05e-102	0.277	0.178	0.029	3.84e-98
B630019A10Rik	0.00e+00	0.705	0.336	0.011	0.00e+00
Tes	6.89e-257	0.590	0.405	0.062	1.29e-252
Izumo1r	4.81e-231	0.670	0.229	0.009	9.01e-227
Itgb2	1.77e-100	0.312	0.511	0.205	3.319e-96
Clec2d	1.42e-285	0.727	0.547	0.119	2.66e-281
Sesn3	5.59e-104	0.280	0.308	0.087	1.05e-99
Klf2	5.09e-268	1.111	0.343	0.040	9.53e-264

Non-differentially expressed genes (Gene set #1) [Sox13–Ppp1r16b]

Differentially expressed genes (Gene set #2) [Igfbp4–Klf2]

DP$_{CD69+}$/SP versus DP$_{small}$

Figure EV3.

© 2022 The Authors

Figure EV4. Workflow used for scRNAseq analysis of DN3 $\gamma\delta^-$ and $\gamma\delta^{int}$ cells from *Lat*$^{-/-}$ thymus.

A Flow cytometry plots showing EGFP and surface TCR $\gamma\delta$ expression in DN1, DN2, DN3, and DN4 subsets from *Trdc-H2BEGFP* (top panels) and *Lat*$^{-/-}$ × *Trdc-H2BEGFP* (bottom panels) mice. Note that the TCR $\gamma\delta^+$ cells corresponding to the DN1 stage correspond to mature $\gamma\delta$ T cells that present a CD127^{hi} phenotype and they were thus excluded from the analysis.

B Schematic representation of the workflow used for scRNAseq analysis of DN3 $\gamma\delta^-$ and DN3 $\gamma\delta^{int}$ cells from thymus (n = 30) of *Lat*$^{-/-}$ × *Trdc-H2BEGFP* mice and the generation of Dataset 3. Two single-cell 3' gene expression libraries containing 18,000 thymoctes and 500 spike-in B cells were constructed and individually sequenced.

C FACS plots showing the gating strategy used to sort DN3 $\gamma\delta^-$ and DN3 $\gamma\delta^{int}$ cells from *Lat*$^{-/-}$ × *Trdc-H2BEGFP* mice. Numbers indicate percentages of cells found in each of the specified gates.

© 2022 The Authors

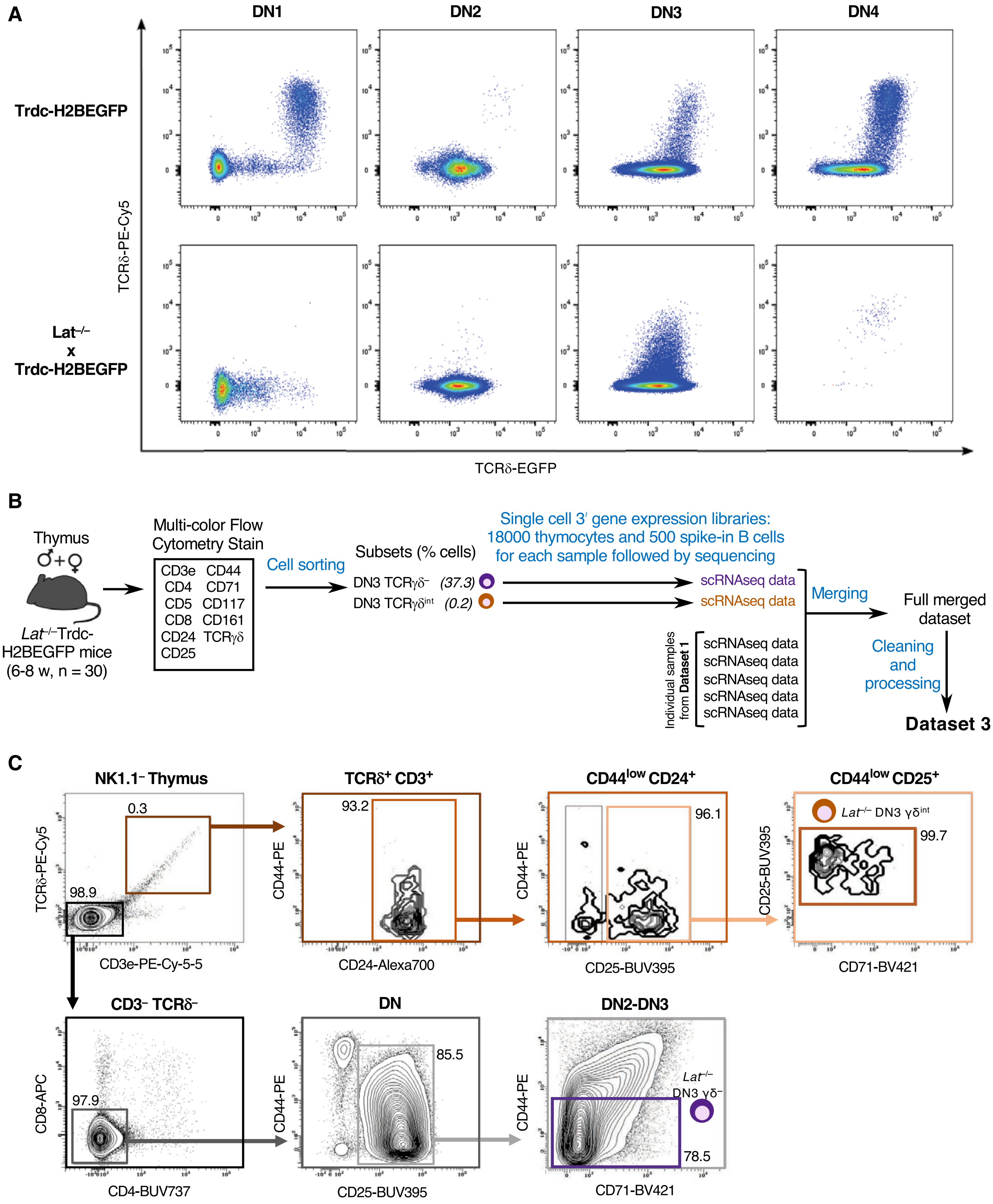

Figure EV4.

 © 2022 The Authors
