# Supplementary material for: Single‐cell transcriptomics uncovers an instructive T‐cell receptor role in adult γδ T‐cell lineage commitment
Source: EMBO J. 2022 Feb 7;41(5):e110023. doi: 10.15252/embj.2021110023 (PMC8886544; doi:10.15252/embj.2021110023)
Supplement: Supplementary file 3 [file EMBJ-41-e110023-s001.pdf]

THE EMBO JOURNAL

Single-cell transcriptomics uncovers the instructive role of the TCR in adult T-cell lineage commitment

Sara Scaramuzzino, Delphine Potier, Robin Ordioni, Pierre Grenot, Dominique Payet-Bornet, Hervé Luche, and Bernard Malissen,

DOI: 10.15252/embj.2021110023

Corresponding author(s): Bernard Malissen (bernardm@ciml.univ-mrs.fr) , Dominique Payet-Bornet (payet@ciml.univ-mrs.fr), Hervé Luche (herve.luche@inserm.fr)

Review Timeline:

Submission Date:	24th Oct 21
Editorial Decision:	10th Dec 21
Revision Received:	18th Dec 21
Accepted:	3rd Jan 22

Editor: Karin Dumstrei

Transaction Report:

(Note: With the exception of the correction of typographical or spelling errors that could be a source of ambiguity, letters and reports are not edited. Depending on transfer agreements, referee reports obtained elsewhere may or may not be included in this compilation. Referee reports are anonymous unless the Referee chooses to sign their reports.)

Hi Bernard,

Thank you for submitting your manuscript to The EMBO Journal. Your study has now been seen by three referees and their reports are provided below.

As you can see from the comments, the referees find that the analysis is well done. Both referees #1 and 2 finds the study interesting and supports publication here without further revisions. Referee #3 is more hesitant if we gain enough new insight.

Having looked at everything, I do find the analysis insightful and that makes a big step forward. I would therefore like to invite you to submit a revised version. There are not many specific points to address - we should discuss if the manuscript should be considered as a resource as suggested by referee #3. I think that I would leave the MS as an article but let me know what you think. Referee #3 raises two points (see below) that would be good to discuss further via email or a video call.

When preparing your letter of response to the referees' comments, please bear in mind that this will form part of the Review Process File, and will therefore be available online to the community. For more details on our Transparent Editorial Process, please visit our website: https://www.embopress.org/page/journal/14602075/authorguide#transparentprocess

We generally allow three months as standard revision time. As a matter of policy, competing manuscripts published during this period will not negatively impact on our assessment of the conceptual advance presented by your study. However, we request that you contact the editor as soon as possible upon publication of any related work, to discuss how to proceed.

I thank you for the opportunity to consider your work for publication.

PS I have attached a document with helpful tips on how to prepare the revised version.

with best wishes

Karin

Karin Dumstrei, PhD
Senior Editor
The EMBO Journal

Guide For Authors: https://www.embopress.org/page/journal/14602075/authorguide

The revision must be submitted online within 90 days; please click on the link below to submit the revision online before 10th Mar 2022.

https://emboj.msubmit.net/cgi-bin/main.plex

Referee #1:

Scaramuzzino et al uses single cell transcriptomics to build on previous studies aimed at dissecting molecular requirements for the divergence of the alpha-beta and gamma-delta T lineages during development in mouse thymus. In doing so, they performed careful single cell transcriptomics on early double negative intermediates of both lineages in order to define the relevant developmental intermediates and expression signatures that define them. Having defined an expression signature for gamma-delta lineage commitment, they then assessed its dependence on TCR signaling by elimination of the critical signaling adaptor LAT. Importantly, LAT loss eliminated the expression signature linked to gamma-delta lineage commitment. This speaks to the two potential explanations for the role of the TCR in lineage commitment as follows. If the stochastic model were correct, then a pre-existing gamma-delta lineage signature would be expected to be present even in the absence of TCR signals, while the instructional model predicts that this signature is TCR dependent. The findings in this manuscript clearly support the latter. The study is well designed, executed, and interpreted, and provides definitive support to instruction for the mode of action of the TCR in gamma-delta lineage commitment.

Referee #2:

The work by Scaramuzzino et al. revisits an important checkpoint during T cell development, which follows the successful rearrangement of a T cell receptor (TCR)-beta chain to form a pre-TCR or TCR-gamma and TCR-delta chains to form a gdTCR-bearing T cell. These rearrangement outcomes have been posited to either reaffirm a pre-commitment lineage outcome for either an abT-lineage or a gdT-lineage, or on the other hand, the rearrangement outcomes themselves give rise to differentiation signaling events that instruct ab vs gd T-lineage outcomes.

The elegant approach taken by the authors makes use of a LAT-deficient mouse model, in which if preprogramming to the gdT cell lineage was to occur, then it would lead to the production of a rearranged gdTCR in these cells, however, in the absence of LAT, the TCR signals would not be transmitted and thus a transcriptomic analysis would reveal whether gdTCR+ cells had already been programmed to initiate gdT cell lineage differentiation or not.

The answer obtained is strikingly clear and consistent with the notion that gdT cell outcomes are dependent on strong TCR signaling, and that the DN3 stage can be a point of lineage bifurcation. With this in mind, the authors should also make reference to earlier work that showed a similar conclusion, Ciofani et al. (Immunity. 2006 Jul;25(1):105-16), by using RAG-deficient cells and then providing a TCRb or TCRgd to ask a similar question.

Nevertheless, the current work beautifully illuminates the transcriptional network that gdTCR vs preTCR induce, including unique and shared pathways, which is an important new insight. Another important new insight is the notion that gdT cell differentiation shares gene pathways with DP to SP abT cell differentiation.

Referee #3:

Single-cell transcriptomics uncovers the instructive role of the TCR in γδ T-cell lineage Commitment

In this manuscript, Scaramuzzino et al make extensive use of single-cell RNA-sequencing to investigate early events in thymocyte development in adult mice. The paper is well written, and the data are of high quality. Moreover, evidence that confirms or supports previously reported events in thymocyte development are presented.

However, although this is a well-executed piece of work, it appears better suited to being a resource paper/study. The novelty aspect of the paper is quite low as most of the data build on previously published work and ideas. Indeed, I suspect the authors are already aware of this, which is presumably why the title focuses on the instruction vs stochastic/selection models of commitment to the γδ T cell lineage, even though this is addressed only near the end of the manuscript.

To focus on this issue, the authors overplay the uncertainty of which model (instruction or stochastic/selection) of commitment to the γδ lineage is correct. Papers referenced in the manuscript from 2005 (with many subsequent confirmations), very nicely demonstrate that lineage commitment to both lineages is instructional, based on strength of signal delivered by whatever TCR complex (preTCR, TCRγδ, or even TCRαβ) a cell expresses on the cell surface during the early DN thymocyte stages. This has shown for the vast majority of developing γδ T cells that the stochastic/selection model is not relevant. The only (possible) exceptions, which is acknowledged by the authors, are certain T cell subsets that are committed in the thymus to make IL-17 (see specifically work from the Kang group on Vγ4+ γδ17 cells).
However, the approach used to assess the relevance of the stochastic/selection model to γδ17 cell development has a major flaw; namely that this study is on adult thymocytes from which γδ17 cells do NOT develop (see many papers - e.g. from the Prinz lab). The γδ17 cells in the adult thymus are thus not the consequence of recent developmental events, having developed many weeks earlier during the perinatal stages. Thus, the absence of any γδ17 cell signature in the LAT-/- DN cells (from adult mice) cannot be taken as evidence that rules out the stochastic/selection model for the development of these cells.

In addition to the above point, the authors also do not address the idea that γδ17 cells may stem from a different DN precursor that is not a DN3 cell. The Kang group specifically focus on DN1d/e cells, and describe a "SOXPRO" subset that they claim directly gives rise to Vγ4+ γδ17 cells. It is not clear whether these subsets (which will be very rare) could be assessed using the approach described in the study. Both points would need to be addressed if the current claims of the manuscript are to be supported.

Letter of response to the Referees' comments

We are grateful to the Reviewers for the time and consideration shown to our manuscript. We are also gratified by the very positive remarks about our paper of both Referees #1 and #2.

All changes in response to the Referee #2 and #3 are highlighted in yellow in the revised manuscript. We also took the opportunity of preparing a revised manuscript to correct a typo in Figure 6 and Figure EV1.

As requested by Referee #2, we added the reference describing the work of Ciofani et al. (Ciofani et al., 2006). They used culture-derived RAG-deficient DN cells retrovirally transduced with either TCR b chain or a combination of TCR gd chains and by growing them on OP9-DL1 cells asked under in vitro conditions a rather similar question to the one we addressed in vivo.

Referee #3 brought up a number of general points and we have addressed them as described below.

1/ We would like to respectfully point out that we stated on pages 4 and 5 of our manuscript that 'the fate of IL17-producing (T γδ 17) γδ T cells that arise during fetal life appears determined prior to TCR gene rearrangement, suggesting that some γδ T cell subsets might develop according to a precommitment model (Melichar et al., 2007; Spidale et al., 2018)'. Therefore, we are well aware of the work of the laboratory of Joonsoo Kang. At no moment, we questioned Kang's data showing that fetal DN thymocytes lacking TCR expression but expressing high levels of IL-7Ra or the high mobility group box transcription factor Sox13 were predisposed to becoming γδ T cells. Accordingly, we have systematically used 'adult thymus' instead of 'thymus' in our original manuscript (overlined in blue in the revised manuscript) to stress that the reported findings solely apply to adult thymus. Due to the specified size limitation, we had to remove the word 'adult' from our title. We will very

much appreciate if the Editors allow us to add it back (Single-cell transcriptomics uncovers the instructive role of the TCR in **adult** γδ T-cell lineage commitment). In such way, the fact that our statement on the instructive model applies to adult and only to adult thymus will be crystal clear from the very beginning.

2/ Note that for the experiment shown in Figure 1 we had to start with 50 adult mouse thymus to obtain at the end enough DP- and SP- depleted cells to proceed to sorting and scRNAseq analysis. Attempting the same experiment on fetal mouse thymus will have needed to extract and process at least 300 thymus over less than two hours to preserve the quality of the cells to be subjected to scRNAseq analysis, a condition that remains technically not tractable. Therefore, our present approach cannot be used to assess fetal γδ17 cell development. Contrary to Reviewer #3 statement, at no moment our approach was thus intended to assess the relevance of the stochastic/selection model to fetal γδ17 cell development.

3/ We would like to comment next on the following points raised by Reviewer #3

'However, the approach used to assess the relevance of the stochatic/selection model to γδ17 cell development has a major flaw; namely that this study is on adult thymocytes from which γδ17 cells do NOT develop (see many papers - e.g. from the Prinz lab). The γδ17 cells in the adult thymus are thus not the consequence of recent developmental events, having developed many weeks earlier during the perinatal stages. Thus, the absence of any γδ17 cell signature in the LAT-/- DN cells (from adult mice) cannot be taken as evidence that rules out the stochastic/selection model for the development of these cells.'

As precisely specified in paragraph 'Identifying the transcriptional signatures induced by the pre-TCR and γδ TCR in adult thymus', the γδ signature we defined is NOT a γδ17 cell signature but a generic γδ signature and in turn we used it to conclude on the absence of a

generic γδ cell signature in the LAT$^{-/-}$ DN cells from adult mice. Along the same line and based on a wealth of studies including an elegant mouse model from Imo Prinz (Sandrock et al., 2018), we are well aware that γδ17 cells do NOT develop in adult thymus and therefore it will have been ludicrous to probe for a γδ17 cell signature in adult thymus. The presence of γδ17 cells in adult thymus is thus thought to be due to self-renewal and/or recirculation from the periphery back into the adult thymus. Although it was not the focus of our study, we would like, however, to point out that our scRNA analysis of adult thymus revealed the presence of γδ17 cells among immature DN4 γδ T cells. A finding congruent with a comprehensive study recently published in The EMBO Journal (Sagar et al., 2020) and comparing on the basis of scRNAseq γδ T-cell functional diversification in fetal and adult thymus.

Finally considering that TCR transgenic mouse models are mentioned by Reviewer #3, we would like to stress that in the case of DN cells, the expression of TCR αβ dimers via transgenesis does not accurately mimic the effect of physiological pre-TCR expression (Baldwin et al., 2005). In contrast, our study aimed at probing the developmental events occurring in a model as close as possible of physiological conditions.

References

Baldwin, T.A., Sandau, M.M., Jameson, S.C., and Hogquist, K.A. (2005). The timing of TCR alpha expression critically influences T cell development and selection. J Exp Med *202*, 111-121.

Ciofani, M., Knowles, G.C., Wiest, D.L., von Boehmer, H., and Zuniga-Pflucker, J.C. (2006). Stage-specific and differential notch dependency at the alphabeta and gammadelta T lineage bifurcation. Immunity *25*, 105-116.

Melichar, H.J., Narayan, K., Der, S.D., Hiraoka, Y., Gardiol, N., Jeannet, G., Held, W., Chambers, C.A., and Kang, J. (2007). Regulation of gammadelta versus alphabeta T lymphocyte differentiation by the transcription factor SOX13. Science *315*, 230-233.

Sagar, Pokrovskii, M., Herman, J.S., Naik, S., Sock, E., Zeis, P., Lausch, U., Wegner, M., Tanriver, Y., Littman, D.R., and Grun, D. (2020). Deciphering the regulatory landscape of fetal and adult gammadelta T-cell development at single-cell resolution. Embo J *39*, e104159.

Sandrock, I., Reinhardt, A., Ravens, S., Binz, C., Wilharm, A., Martins, J., Oberdorfer, L., Tan, L., Lienenklaus, S., Zhang, B., *et al.* (2018). Genetic models reveal origin, persistence and non-redundant functions of IL-17-producing gammadelta T cells. J Exp Med *215*, 3006-3018.

Spidale, N.A., Sylvia, K., Narayan, K., Miu, B., Frascoli, M., Melichar, H.J., Zhihao, W., Kisielow, J., Palin, A., Serwold, T., *et al.* (2018). Interleukin-17-Producing gammadelta T Cells Originate from SOX13(+) Progenitors that Are Independent of gammadeltaTCR Signaling. Immunity *49*, 857-872 e855.

Dear Bernard,

Thank you for submitting your revised manuscript to The EMBO Journal. I have now had a chance to take a careful look at everything and all looks good!

I am therefore very pleased to accept the manuscript for publication here.

With best wishes

Karin

Karin Dumstrei, PhD
Senior Editor
The EMBO Journal

Please note that it is EMBO Journal policy for the transcript of the editorial process (containing referee reports and your response letter) to be published as an online supplement to each paper. If you do NOT want this, you will need to inform the Editorial Office via email immediately. More information is available here:
https://www.embopress.org/page/journal/14602075/authorguide#transparentprocess

Your manuscript will be processed for publication in the journal by EMBO Press. Manuscripts in the PDF and electronic editions of The EMBO Journal will be copy edited, and you will be provided with page proofs prior to publication. Please note that supplementary information is not included in the proofs.

Please note that you will be contacted by Wiley Author Services to complete licensing and payment information. The 'Page Charges Authorization Form' is available here: https://www.embopress.org/pb-assets/embo-site/tej_apc.pdf

Should you be planning a Press Release on your article, please get in contact with embojournal@wiley.com as early as possible, in order to coordinate publication and release dates.

If you have any questions, please do not hesitate to call or email the Editorial Office. Thank you for your contribution to The EMBO Journal.

** Click here to be directed to your login page: https://emboj.msubmit.net

EMBO PRESS

YOU MUST COMPLETE ALL CELLS WITH A PINK BACKGROUND

PLEASE NOTE THAT THIS CHECKLIST WILL BE PUBLISHED ALONGSIDE YOUR PAPER

Corresponding Author Name: MALISSEN Bernard
Journal Submitted to: EMBO JOURNAL
Manuscript Number: EMBOJ-2021-110023R

Reporting Checklist For Life Sciences Articles (Rev. June 2017)

This checklist is used to ensure good reporting standards and to improve the reproducibility of published results. These guidelines are consistent with the Principles and Guidelines for Reporting Preclinical Research issued by the NIH in 2014. Please follow the journal's authorship guidelines in preparing your manuscript.

A- Figures
1. Data
The data shown in figures should satisfy the following conditions:

- the data were obtained and processed according to the field's best practice and are presented to reflect the results of the experiments in an accurate and unbiased manner.
- figure panels include only data points, measurements or observations that can be compared to each other in a scientifically meaningful way.
- graphs include clearly labeled error bars for independent experiments and sample sizes. Unless justified, error bars should not be shown for technical replicates.
- if n< 5, the individual data points from each experiment should be plotted and any statistical test employed should be justified
- Source Data should be included to report the data underlying graphs. Please follow the guidelines set out in the author ship guidelines on Data Presentation.

2. Captions

Each figure caption should contain the following information, for each panel where they are relevant:

- a specification of the experimental system investigated (eg cell line, species name).
- the assay(s) and method(s) used to carry out the reported observations and measurements
- an explicit mention of the biological and chemical entity(ies) that are being measured.
- an explicit mention of the biological and chemical entity(ies) that are altered/varied/perturbed in a controlled manner.

- the exact sample size (n) for each experimental group/condition, given as a number, not a range;
- a description of the sample collection allowing the reader to understand whether the samples represent technical or biological replicates (including how many animals, litters, cultures, etc.).
- a statement of how many times the experiment shown was independently replicated in the laboratory.
- definitions of statistical methods and measures:
  - common tests, such as t-test (please specify whether paired vs. unpaired), simple χ2 tests, Wilcoxon and Mann-Whitney tests, can be unambiguously identified by name only, but more complex techniques should be described in the methods section;
  - are tests one-sided or two-sided?
  - are there adjustments for multiple comparisons?
  - exact statistical test results, e.g., P values = x but not P values < x;
  - definition of 'center values' as median or average;
  - definition of error bars as s.d. or s.e.m.

Any descriptions too long for the figure legend should be included in the methods section and/or with the source data.

In the pink boxes below, please ensure that the answers to the following questions are reported in the manuscript itself. Every question should be answered. If the question is not relevant to your research, please write NA (non applicable). We encourage you to include a specific subsection in the methods section for statistics, reagents, animal models and human subjects.

USEFUL LINKS FOR COMPLETING THIS FORM

http://www.antibodypedia.com
http://1degreebio.org
http://www.equator-network.org/reporting-guidelines/improving-bioscience-research-repo
http://grants.nih.gov/grants/olaw/olaw.htm
http://www.mrc.ac.uk/Ourresearch/Ethicsresearchguidance/Useofanimals/index.htm
http://ClinicalTrials.gov
http://www.consort-statement.org
http://www.consort-statement.org/checklists/view/32-consort/66-title
http://www.equator-network.org/reporting-guidelines/reporting-recommendations-for-tum
http://datadryad.org
http://figshare.com
http://www.ncbi.nlm.nih.gov/gap
http://www.ebi.ac.uk/ega
http://biomodels.net/
http://biomodels.net/miriam/
http://jjj.biochem.sun.ac.za
https://osp.od.nih.gov/biosafety-biosecurity-and-emerging-biotechnology/
http://www.selectagents.gov/

B- Statistics and general methods

Please fill out these boxes (Do not worry if you cannot see all your text once you press return)

1.a. How was the sample size chosen to ensure adequate power to detect a pre-specified effect size?	The sample sizes were chosen from past knowledge on good sample size to ensure adequate power. Sample sizes are always indicated in figure legends or related "Materiels and Methods" section.
1.b. For animal studies, include a statement about sample size estimate even if no statistical methods were used.	For scRNAseq experiments, we used 50 mice to generate dataset 1, 2 mice to generate dataset2 and 30 mice to generate the two extra samples present in dataset 3. For flow cytometry 6 mice were used per experimental conditions. All mice experiments had to be in line with the rules of our ethical committee and match the 3 Rs rule.
2. Describe inclusion/exclusion criteria if samples or animals were excluded from the analysis. Were the criteria pre-established?	No data where excluded from the analyses.
3. Were any steps taken to minimize the effects of subjective bias when allocating animals/samples to treatment (e.g. randomization procedure)? If yes, please describe.	In all experiments an equal number of male and female were used.
For animal studies, include a statement about randomization even if no randomization was used.	No randomization was used.
4.a. Were any steps taken to minimize the effects of subjective bias during group allocation or/and when assessing results (e.g. blinding of the investigator)? If yes please describe.	Blind group assignment cannot be applied.
4.b. For animal studies, include a statement about blinding even if no blinding was done	Blind group assignment cannot be applied.
5. For every figure, are statistical tests justified as appropriate?	Yes, statistical tests are indicated in the materiel and methods section.
Do the data meet the assumptions of the tests (e.g., normal distribution)? Describe any methods used to assess it.	Yes. For group of cells gene expression comparison non-parametric statistics was used.

Is there an estimate of variation within each group of data?	No , we performed only non-parametrical tests
Is the variance similar between the groups that are being statistically compared?	Not assessed.

C- Reagents

6. To show that antibodies were profiled for use in the system under study (assay and species), provide a citation, catalog number and/or clone number, supplementary information or reference to an antibody validation profile. e.g., Antibodypedia (see link list at top right), 1DegreeBio (see link list at top right).	All the antibodies were commercially available. Their commercial references and their RRID are indicated in appendix table S1.
7. Identify the source of cell lines and report if they were recently authenticated (e.g., by STR profiling) and tested for mycoplasma contamination.	No cell line was used.

* for all hyperlinks, please see the table at the top right of the document

D- Animal Models

8. Report species, strain, gender, age of animals and genetic modification status where applicable. Please detail housing and husbandry conditions and the source of animals.	Mice were kept under SOPF conditions. Age is specified in the Materials and Method section. Males and females were used. The origin and international nomenclature of the mice with a genetic modification are provided.
9. For experiments involving live vertebrates, include a statement of compliance with ethical regulations and identify the committee(s) approving the experiments.	Mice were handled in accordance with national and European laws for laboratory animal welfare and experimentation (European Economic Community Council Directive 2010/63/EU, September 2010).
10. We recommend consulting the ARRIVE guidelines (see link list at top right) (PLoS Biol. 8(6), e1000412, 2010) to ensure that other relevant aspects of animal studies are adequately reported. See author guidelines, under 'Reporting Guidelines'. See also: NIH (see link list at top right) and MRC (see link list at top right) recommendations. Please confirm compliance.	We fully complied with them.

E- Human Subjects

11. Identify the committee(s) approving the study protocol.	Irrelevant
12. Include a statement confirming that informed consent was obtained from all subjects and that the experiments conformed to the principles set out in the WMA Declaration of Helsinki and the Department of Health and Human Services Belmont Report.	Irrelevant
13. For publication of patient photos, include a statement confirming that consent to publish was obtained.	Irrelevant
14. Report any restrictions on the availability (and/or on the use) of human data or samples.	Irrelevant
15. Report the clinical trial registration number (at ClinicalTrials.gov or equivalent), where applicable.	Irrelevant
16. For phase II and III randomized controlled trials, please refer to the CONSORT flow diagram (see link list at top right) and submit the CONSORT checklist (see link list at top right) with your submission. See author guidelines, under 'Reporting Guidelines'. Please confirm you have submitted this list.	Irrelevant
17. For tumor marker prognostic studies, we recommend that you follow the REMARK reporting guidelines (see link list at top right). See author guidelines, under 'Reporting Guidelines'. Please confirm you have followed these guidelines.	Irrelevant

F- Data Accessibility

18: Provide a "Data Availability" section at the end of the Materials & Methods, listing the accession codes for data generated in this study and deposited in a public database (e.g. RNA-Seq data: Gene Expression Omnibus GSE39462, Proteomics data: PRIDE PXD000208 etc.) Please refer to our author guidelines for 'Data Deposition'.<br><br>Data deposition in a public repository is mandatory for:<br>a. Protein, DNA and RNA sequences<br>b. Macromolecular structures<br>c. Crystallographic data for small molecules<br>d. Functional genomics data<br>e. Proteomics and molecular interactions	Done.
19. Deposition is strongly recommended for any datasets that are central and integral to the study; please consider the journal's data policy. If no structured public repository exists for a given data type, we encourage the provision of datasets in the manuscript as a Supplementary Document (see author guidelines under 'Expanded View' or in unstructured repositories such as Dryad (see link list at top right) or Figshare (see link list at top right).	Done. ScRNAseq raw data and raw count were respectively deposited in SRA and in GEO.
20. Access to human clinical and genomic datasets should be provided with as few restrictions as possible while respecting ethical obligations to the patients and relevant medical and legal issues. If practically possible and compatible with the individual consent agreement used in the study, such data should be deposited in one of the major public access-controlled repositories such as dbGAP (see link list at top right) or EGA (see link list at top right).	Irrelevant.
21. Computational models that are central and integral to a study should be shared without restrictions and provided in a machine-readable form. The relevant accession numbers or links should be provided. When possible, standardized format (SBML, CellML) should be used instead of scripts (e.g. MATLAB). Authors are strongly encouraged to follow the MIRIAM guidelines (see link list at top right) and deposit their model in a public database such as Biomodels (see link list at top right) or JWS Online (see link list at top right). If computer source code is provided with the paper, it should be deposited in a public repository or included in supplementary information.	Irrelevant.

G- Dual use research of concern

22. Could your study fall under dual use research restrictions? Please check biosecurity documents (see link list at top right) and list of select agents and toxins (APHIS/CDC) (see link list at top right). According to our biosecurity guidelines, provide a statement only if it could.	No.
